# A Systematic Review of Guided, Parent-Led Digital Interventions for Preadolescent Children with Emotional and Behavioural Problems

**DOI:** 10.1007/s10567-025-00521-x

**Published:** 2025-05-11

**Authors:** Emily Whitaker, Chloe Chessell, Maxwell Klapow, Cathy Creswell

**Affiliations:** 1 Department of Experimental Psychology, University of Oxford, Oxford, UK; 2 Department of Psychiatry, University of Oxford, Oxford, UK; 3 Department of Social Policy and Intervention, University of Oxford, Oxford, UK

**Keywords:** Emotional and behavioural problems, Digital interventions, Children, Parents

## Abstract

Emotional and behavioural problems (EBP) are prevalent amongst children, and guided, parent-led digital interventions offer one method of improving access to effective treatments. This systematic review (PROSPERO: CRD42023484098) aimed to examine the evidence base for, and characteristics of, these types of interventions through a narrative synthesis. Systematic searches were conducted using Medline, EMBASE, PsycINFO, Scopus and Web of Science in January 2024 and February 2025, supplemented with hand searching in March/April 2024 and February 2025. Studies were eligible if they reported outcomes related to preadolescent EBP from a guided, fully parent-led, fully digital intervention. Thirteen studies were eligible, including 2643 children and covering eight interventions (addressing anxiety problems, comorbid anxiety and depression, attention deficit hyperactivity disorder, conduct disorder and disruptive behaviour). Studies included randomised controlled trials and pre-post studies. The QualSyst checklist was used to assess study quality; all studies were rated as good quality. All studies showed statistically significant improvements in the child’s symptoms or interference levels, with small to very large effect sizes immediately post-treatment, and at least medium effect sizes by follow-up, suggesting a promising evidence base. A wide range of intervention characteristics were identified, forming a basis for future intervention development for childhood EBP. However, there was a lack of consistency in how information was reported across studies (such as completion rates) and studies lacked information on parent demographics and key intervention details. Further high quality randomised controlled trials for a wider range of EBP are needed to continue building the evidence base.

**Supplementary Information:**

The online version contains supplementary material available at 10.1007/s10567-025-00521-x.

## Background

Emotional and behavioural problems (EBP) are prevalent amongst children, encompassing mental health conditions such as anxiety problems, mood disorders, attention deficit hyperactivity disorder (ADHD) and conduct disorders (Ogundele, 2018). Worldwide prevalence of EBP is difficult to estimate, but studies have suggested, for instance, 6.5% of children worldwide have an anxiety disorder and 5.7% have a disruptive disorder (Polanczyk et al., 2015). Such difficulties typically emerge early in life, with a peak age of onset of, for example, 5.5 years for anxiety disorders and 9.5 years for ADHD (Solmi et al., 2022). EBP can cause a range of difficulties during childhood, including social (Hukkelberg et al., 2019) and academic impairment (Kremer et al., 2016; Murphy et al., 2015). If left to persist, EBP can have an ongoing negative impact on individuals’ lives into adolescence and adulthood, affecting employment (Egan et al., 2015; Fergusson et al., 2005), and increasing the risk for long-term mental and physical health problems (Kretschmer et al., 2014; Schlack et al., 2021).

Given the early age of onset and negative consequences of childhood EBP, effective intervention during the preadolescent years is essential. A range of treatments are currently available and have been found to be effective in children, including cognitive behavioural therapy (CBT) for anxiety problems (James et al., 2020) and depressive symptoms (Arnberg & Öst, 2014), and parent training programmes for behavioural problems (National Institute for Health and Care Excellence, 2017). However, despite the existence of evidence-based treatments for EBP, children and their families struggle to access them, citing barriers such as long waiting lists and perceived stigma (Baweja et al., 2021; Reardon et al., 2017).

One potential method of increasing access to treatment and overcoming these barriers is through guided, parent-led digital interventions. Brief guided, parent-led interventions (i.e. where a parent learns tools and techniques that they can use at home to help their child; Jewell et al., 2022) are commonly delivered for preadolescents with EBP (Ludlow et al., 2020), have been shown to be effective, and can be delivered in ways that require less therapist time than child-focused approaches (e.g. Sampaio et al., 2018; Sanders et al., 2014; Thirlwall et al., 2013), indicating their potential to help increase access to treatment for preadolescent children with EBP. They can also help to reduce perceived stigma for children and parents; for example, parents have identified not wanting their child to feel as though they have a problem or not wanting other people to know about their child’s difficulties as reasons for not seeking help (Reardon et al., 2020). Delivering interventions directly to the parent, and not requiring the child to attend (and have usual routines disrupted including potentially being taken out of school for treatment) can help overcome these barriers and help ensure treatment is accessed when families first need it.

The benefits of guided parent-led interventions can be further enhanced when the intervention is delivered digitally (Baumel et al., 2017; David et al., 2023). Treatments that are delivered through digital means, such as via the internet, software or a smartphone application (‘app’), have risen in prominence in recent years, particularly following the COVID-19 pandemic (Baweja et al., 2021). Digital interventions are typically shorter in duration (Kambeitz-Ilankovic et al., 2022) and often require less therapist input (Andersson & Titov, 2014) than face-to-face interventions, bringing potential to reduce waiting times to access treatment. Digital interventions also allow families to receive treatment from their own home and in their own time, thereby allowing the intervention to fit around busy lives and further reducing the potential for concerns relating to stigma (McGoron & Ondersma, 2015; Weisenmuller & Hilton, 2021).

To date, there has been a lack of synthesis regarding the characteristics of these treatments for preadolescent children specifically. It is important to focus on preadolescents not only due to most EBP beginning in this age range, but also due to the differing clinical characteristics and treatment needs of preadolescents compared to adolescents (Huefner & Vollmer, 2014; Waite & Creswell, 2014). Identifying the characteristics of guided parent-led digital interventions for preadolescents with EBP is important to provide a foundation for the development of new digital treatments for this population, should the evidence base suggest this is warranted. Almost a decade ago, MacDonell and Prinz (2017) conducted a review of youth and family-focused technology-based interventions and found that the interventions utilised audio components, videos, animations and games; however only a limited set of technical characteristics were coded, and the theoretical basis (e.g. CBT) and therapeutic components of the interventions were not captured. In contrast, Yun et al. (2023) did capture this information in their more recent broad review of digital interventions for children and young people (up to 19 years old) with any type of mental health or physical health problem, but as their review only included papers published between 2018 and 2022, many relevant studies were not included, and only two parent-led interventions were identified. A helpful recent review by Grajdan et al. (2024) focused on parent involvement in digital interventions specifically for anxiety problems highlighted promising findings and a variety of intervention characteristics. However, the broader scope (including e.g. interventions where up to half of the intervention could be directed to the child and blended approaches incorporating both face to face and digital support) limited specific conclusions about guided, parent-led digital approaches.

To our knowledge, there are currently no systematic reviews that focus exclusively on guided, parent-led digital interventions for preadolescents with EBP. Moreover, there is a lack of knowledge regarding what such interventions typically involve, with regards to their technical functions and therapeutic components. Given the need for early treatment, a review of the available interventions targeted at this specific age range is warranted to drive future research and practice. Specifically, our research questions were:

1. What is the evidence base for guided, parent-led digital interventions for preadolescent children with emotional and behavioural problems?
2. What are the characteristics of guided, parent-led digital interventions for preadolescent children with emotional and behavioural problems?

## Methods

The methodological approach taken in this review was guided by the Cochrane Handbook (Higgins & Green, 2008). We followed the Preferred Reporting Items for Systematic Reviews and Meta-Analyses (PRISMA) guidelines (Page et al., 2021) and the Synthesis Without Meta-analysis (SWiM) guidelines (Campbell et al., 2020). The PRISMA and SWiM checklists can be found in Appendices 1 and 2. The review was prospectively registered on the International Prospective Register of Systematic Reviews (PROSPERO; CRD42023484098) and changes made to the original protocol are outlined in Appendix 3.

### Search Strategy

Scoping searches were undertaken to identify key papers and to refine the search strategy, and input on the search terms was received from University of Oxford Bodleian librarians.

The following databases were searched on 2nd January 2024 for peer-reviewed articles and grey literature: PsycINFO (Ovid), MEDLINE (Ovid), Embase (Ovid), Web of Science (all databases) and Scopus. Search terms related to preadolescents, parents, guidance, digital interventions, and emotional and behavioural problems (Table 1 shows examples of the search terms used). Boolean operators, proximity operators and Medical Subject Headings (MeSH terms) were used and adapted as appropriate to each database. The full search strategy for each database can be seen in Appendix 4. An updated search, using the same five databases and with the same search strategy, was performed on 27th February 2025.

**Table 1:** Examples of terms used in the database searches

Search category	Examples of search terms
Preadolescent	*Preadolescent* or *child* or *young* or *paediatric*
Parent	*Parent* or *carer* or *caregiver* or* family*
Guidance	*Guided or support or led or assist*
Digital	*Digital* or *remote* or *online* or* internet*
Intervention	*Intervention* or *treatment* or *therapy* or* programme*
Problem	*“emotional problem”* or *“behavioural problem”* or *internalising* or *externalising* or *anxiety* or* conduct*

Where possible, filters were applied to restrict the results to human studies and studies written in English. No other filters were used. No date restrictions were applied; all study designs were eligible for inclusion; and results were not limited to published or peer-reviewed articles, thereby reducing publication bias (Paez, 2017).

Initial hand searching took place between March and April 2024 to identify any additional studies. Forward and backward citation searching of all included studies was conducted, and over 30 previous reviews in relevant areas were checked. Google Scholar was used for the forward citation searching. Further forward citation searching was undertaken in February 2025.

### Inclusion and Exclusion Criteria

Studies were eligible for inclusion in the review if: (i) the full text was available in English; (ii) the study evaluated the effect of a psychological intervention in which a parent is supported to deliver the treatment by a facilitator; (iii) all aspects of the intervention were digital and targeted at least one child EBP; (iv) the participants were parents/carers of children with a mean age of 4–12 years; and (v) included quantitative data from at least pre- and post-intervention outcome measures of symptoms/interference related to the child’s EBP.

Studies were not included in the review if: (i) the intervention involved face-to-face contact with the facilitator; (ii) the intervention was not delivered directly via the parent; (iii) the intervention was delivered directly to the child; (iv) the intervention did not feature any guidance by a facilitator; (v) the study was a systematic review, meta-analysis or protocol; or (vi) the study only reported previously published data.

A summary of the criteria in PICO format (Population, Intervention, Comparator, Outcome) can be found in Table 2, and a more detailed version of the criteria can be found in Appendix 5.

**Table 2:** Inclusion and exclusion criteria

	Inclusion criteria	Exclusion criteria
Population	Children aged 4–12 years old (to maximise inclusion samples with an age range of up to 14 years old were included as long as the mean age fell within this range). Children should be formally diagnosed with an EBP, be displaying elevated symptoms of an EBP problem according to an established measure, or be considered by a clinical team to have an EBP	Samples where the upper age range of the participants was 15 years old or above Samples with the mean age outside of 4–12 years old
Intervention	Digital interventions for treating children’s EBP. For this review, ‘digital’ referred to a website, app or software. The intervention content should be fully digital. Interventions should be solely delivered to the child’s parent and must be guided by a facilitator	Interventions that were purely telehealth, featured any contact with the child, featured any face-to-face elements, or included any physical materials were excluded
Comparator	No comparator was required	For studies that did use a comparator, there were no restrictions on the type
Outcome	Outcomes should relate to the child’s EBP and an established/standardised measure should be used. Pre- and post-intervention measures should be reported	Outcomes that only relate to the parent’s mental health or wellbeing rather than the child’s EBP

### Study Selection

The results from the electronic database searches were initially uploaded into the reference manager Endnote, and then into the systematic review software Covidence where duplicates were automatically removed. Further duplicates were identified manually. All titles and abstracts were screened by one reviewer (EW) against the eligibility criteria, and a second reviewer (MK) independently screened 20% of the titles/abstracts from the initial database search. Studies were put through to the full text stage if they appeared to be relevant, or if there was not enough information in the title/abstract to fully assess the relevance. Full texts were then retrieved and 100% were independently screened by the two reviewers (EW and MK) against the eligibility criteria. For the updated search performed in February 2025, all screening was conducted by a single reviewer (EW). During the full text stage, articles were excluded in a stepped fashion and all exclusion reasons were recorded (see Appendix 6 for the stepped exclusion list and Appendix 7 for all articles excluded at the full text stage). All disagreements were resolved through discussion, with the involvement of CCh and CCr when necessary. Percentage agreement and Cohen’s kappa (Landis & Koch, 1977) were calculated at both screening stages to measure inter-rater reliability. Agreement between the two reviewers was 84% (*k* = 0.26) at the title/abstract stage and 92% (*k* = 0.71) at the full text stage.

### Data Extraction

For all studies that met the inclusion criteria, data were extracted from the full texts and recorded in a data extraction form created in Microsoft Excel. This form was piloted and refined prior to the database search commencing with a sample of papers. Extracted data included study characteristics, intervention therapeutic components and technical functionalities, participant details and intervention outcomes. Data were extracted by one reviewer (EW) and cross-checked for accuracy by a second reviewer (MK).

For intervention components and functionalities, a coding system was developed and each included intervention was rated against it. The coding system was informed by previous systematic reviews and was developed by listing characteristics of the interventions in any of the included studies, and then rating each intervention against the list. Other research articles featuring the included interventions were also examined to extract further details about the intervention characteristics. In addition, all study authors and/or intervention developers were contacted to confirm the accuracy of the coding applied to the intervention. Individuals were contacted via email and a follow-up email was sent if there was no reply after two weeks. If there was no reply after a further two weeks, the coding system remained how it was. When contacting authors, other key missing information about their study was also requested, for example, missing participant demographics or data needed to calculate Cohen’s *d*. Replies were received from 53.8% of authors, covering 62.5% of the included interventions.

For intervention outcomes, Cohen’s *d* was reported for all studies at the pre-intervention, post-intervention and follow-up period (if applicable). We chose to convert other effect sizes to Cohen’s *d* in order to allow ease of comparison across study outcomes. When Cohen’s *d* was not reported by the study authors, the review authors calculated it using Psychometrica (Lenhard & Lenhard, 2017). Standard errors were converted to standard deviations for several studies, following the formula given in Chapter 6 in the Cochrane Handbook (Higgins & Green, 2008). Effect sizes were interpreted according to Cohen’s (2013) conventions. If follow-up periods were reported in a separate study, the results were pooled for data extraction. Only the primary outcome measure was extracted. If no outcomes were explicitly identified as being primary, the first reported outcome measure was taken as being the primary one, and we prioritised measures of symptoms or interference/functioning, rather than diagnostic severity, since this was the most common form of primary outcome measure.

### Quality Assessment

The Checklist for Assessing the Quality of Quantitative Studies (QualSyst; Kmet et al., 2004) was used to evaluate the quality of the included studies. QualSyst was chosen as it is suitable for use with both randomised and non-randomised studies. It contains 14 items, relating to areas such as the study design, participant demographics, and the study outcomes. Each item is scored out of two, with answering ‘yes’ to an item resulting in the maximum score of two points, ‘partial’ giving one point, and ‘no’ giving zero points. This gives a total possible score of 28. Two reviewers (EW and MK) independently evaluated each included study using the checklist. Any disagreements or queries were resolved via discussion and the input of a third reviewer (CCh/CCr) if necessary. Scores were given as a percentage, as not all items on the checklist were applicable for each study included in this review. Studies were not excluded from the review upon the basis of their quality score.

### Data Synthesis

Due to the heterogeneity across the included studies (in terms of the populations being targeted, the study designs and the interventions themselves), a meta-analysis was not conducted. Instead, we conducted a narrative synthesis of the results (Popay et al., 2006). The key areas synthesised in this review were the study characteristics, study samples, interventions, outcomes and quality of the included studies. Each of these areas were briefly synthesised as a whole, across all included studies, before being grouped into studies/interventions focused on emotional problems and behavioural problems. These groupings were chosen as we anticipated differences in the key areas depending on which problem type the intervention was focused on. To complement the narrative synthesis, data were also presented in tabular format. Tables grouped studies according to whether the focus was on an emotional problem or a behavioural problem, and then grouped further by intervention, with the intervention being listed alphabetically.

## Results

### Study Selection

**Fig. 1:**
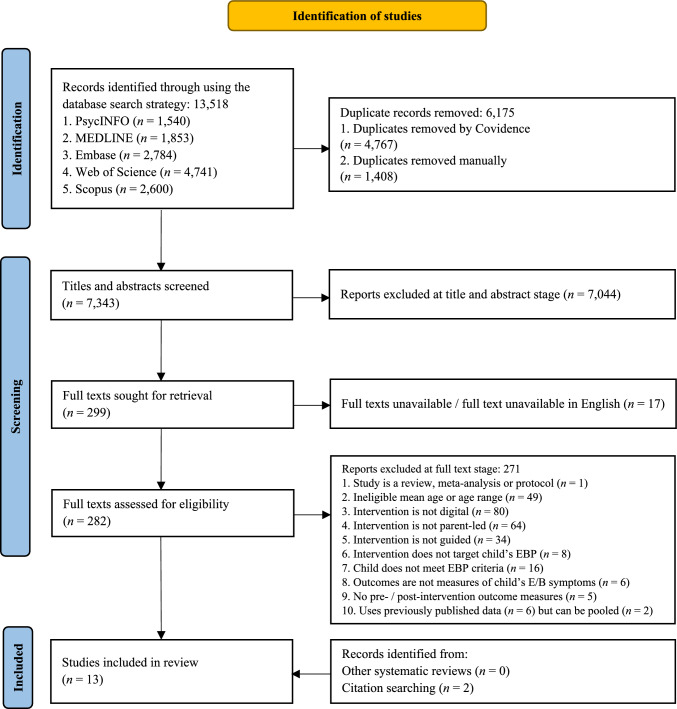
Flow of selection and exclusion of studies

A total of 13,518 records were identified through the electronic databases, across both the original search (January 2024) and the updated search (February 2025). Of these, 6175 records were removed as duplicates, resulting in the titles and abstracts of 7343 records being screened. After initial screening, 7044 records were excluded as ineligible. Subsequently, full texts in English were successfully retrieved for 282 records and these were subjected to full text screening. Of these 282 records, 11 met the inclusion criteria and were included in the narrative synthesis. A further two eligible studies were identified from handsearching, giving a total of 13 studies in the narrative synthesis. A PRISMA-style flow chart is displayed in Fig. 1. Further details around exclusion reasons at the full text stage can be found in Appendix 7, along with examples of studies that may appear to meet the eligibility criteria but were excluded.

In addition to the 13 studies included in the review, three studies (Högström et al., 2015; A. Sourander et al., 2018; S. Sourander et al., 2024) were identified as providing longer term follow-up data relating to included studies. Data from these studies were included in the synthesis of the study outcomes.

One paper (Dadds et al., 2019) included two studies with different, independent samples. For the purposes of the narrative synthesis, this paper will be referred to as one ‘study’ unless otherwise indicated. Any relevant differences between the two studies were extracted and reported upon in the tables below. In another paper (Green et al., 2023) not all participants fully met the criteria for the review, but as data were reported for a subset that did meet all inclusion criteria these were included in this review.

### Study Characteristics

Table 3 presents a summary of the key characteristics of the eligible studies. The 13 included studies were all conducted relatively recently, from 2012 to 2024. Six studies focused on emotional problems (Creswell et al., 2024; Donovan & March, 2014; Green et al., 2023; Hill et al., 2022; Mazenc, 2021; Poetar et al., 2024) and seven focused on behavioural problems (Dadds et al., 2019; Enebrink et al., 2012; Franke et al., 2020; Ghaderi et al., 2018; A. Sourander et al., 2016; A. Sourander et al., 2022, S. Sourander et al., 2022). Almost all of the studies were published in peer-reviewed journals, with just one study (Mazenc, 2021) in an unpublished doctoral thesis.

**Table 3:** Key characteristics of the included studies

Author (date)	Country	Study design	Recruitment setting	Digital intervention	Target problem	Full sample	Child age range
Mazenc (2021)	Canada	Pre-post study	Clinic and community	ACE	Emotional: anxiety	91	7–12 years
Donovan and March (2014)	Australia	RCT	Community	BRAVE-ONLINE	Emotional: anxiety	52	3–6 years
Creswell et al. (2024)	UK	RCT	Clinic	OSI	Emotional: anxiety	444	5–12 years
Green et al. (2023)	UK	Pre-post study	Clinic	OSI	Emotional: anxiety	49	8–9 years
Hill et al. (2022)	UK	Pre-post study	School	OSI	Emotional: anxiety	23	7–12 years
Poetar et al. (2024)	Romania	RCT	Community	ParentKIT	Emotional: transdiagnostic^a^	42	6–14 years
Dadds et al. (2019)^b^	Australia	RCT	Clinic	AccessEI	Behavioural: conduct	133/73	3–9/3–14 years
Enebrink et al. (2012)	Sweden	RCT	Community	iComet	Behavioural: conduct	104	3–12 years
Ghaderi et al. (2018)	Sweden	RCT	Community	iComet	Behavioural: conduct	231	10–13 years
A. Sourander et al. (2016)	Finland	RCT	Clinic	SFSW	Behavioural: disruptive behaviour	464	4 years
A. Sourander et al. (2022)	Finland	Pre-post study	Clinic	SFSW	Behavioural: disruptive behaviour	832	4 years
S. Sourander et al. (2022)	Finland	Pre-post study	Clinic	SFSW	Behavioural: disruptive behaviour	50	3–8 years
Franke et al. (2020)	New Zealand	RCT	Community	Triple P Online	Behavioural: ADHD	53	3–4 years

*Digital intervention abbreviations: ACE* Anxiety treatment for Children through online Education*; OSI* Online Support and Intervention for child anxiety; *SFSW* Strongest Families Smart Website.^a^Poetar et al.(2024): ParentKIT is a transdiagnostic intervention targeting both anxiety and depressive symptoms. ^b^Dadds et al. (2019): the forward slash reflects Study 1/Study 2. Where no forward slash is used, the information was the same across both studies

Of the six studies that focused on emotional problems, half were randomised controlled trials (RCTs; Creswell et al., 2024; Donovan & March, 2014; Poetar et al., 2024) whilst the others were non-randomised pre-post designs. All studies were conducted in high income countries, namely the UK (*n* = 6), Canada (*n* = 1), Australia (*n* = 1) and Romania (*n* = 1). Recruitment settings varied, with community, schools and clinics being used to recruit families. Total sample sizes ranged from 23 (an uncontrolled case series conducted in a university clinic; Hill et al., 2022) to 444 (an RCT based across multiple child mental health services; Creswell et al., 2024). The comparators used in the RCTs were usual treatment (Creswell et al., 2024) and a waitlist (Donovan & March 2014; Poetar et al., 2024).

Of the seven studies focused on behavioural problems, five were RCTs (Dadds et al., 2019; Enebrink et al., 2012; Franke et al., 2020; Ghaderi et al., 2018; A. Sourander et al., 2016). and the remaining two were non-randomised pre-post designs. All seven studies took place in high income countries: Finland (*n* = 3) Sweden (*n* = 2), Australia (*n* = 1) and New Zealand (*n* = 1). Families were recruited from clinics and the community. The smallest sample size was 50 (a feasibility study; S. Sourander et al., 2022) and the largest sample size was 832 (an implementation study using a pre-post design; A. Sourander et al., 2022a). The comparators used in the five RCTs were waitlists (Enebrink et al., 2012; Franke et al., 2020), face-to-face versions of the digital intervention (Dadds et al., 2019; Ghaderi et al., 2018), and an educational control (A. Sourander et al., 2016). Additionally, in their implementation study, A. Sourander et al. (2022) compared the intervention as used in routine practice with the outcomes from one of the included RCTs (A. Sourander et al., 2016).

### Sample Characteristics

Table 4 presents a summary of the sample characteristics of the included studies. A more detailed version of the table, including socio-demographic information and child diagnostic criteria, can be found in Appendix 8. Sample characteristics are reported for the intervention group only, rather than for any comparators. Children in the included studies were from across the eligible age range of 4–12 years, with three studies extending up to 14 years (in accordance with the inclusion criteria, the mean child age for these studies was below 12 years). Child gender was reported in all but one study (Ghaderi et al., 2018). In several studies, the child demographics were not available for the sample of interest: one study only provided child demographics for the completer sample (Mazenc, 2021); one RCT only provided age/gender for the whole sample, rather than broken down by arm (Donovan & March, 2014); one study only provided child demographics for the whole sample rather than the subset with an anxiety problem (Green et al., 2023). With regards to child ethnicity, related information was reported in some form in seven studies (54%), with three of these studies focusing on nationality rather than ethnicity.

**Table 4:** Sample characteristics of the included studies (intervention arm only, unless stated in the footnote)

Author (date); intervention	Sample size (intervention)	Mean child age in years (SD)	Child gender (% female)	Child ethnicity (% White)	Mean parent age in years (SD)	Parent gender	Parent ethnicity (% White)
Mazenc (2021)^a^; ACE	93 / 47	9.38 (1.67)	57.4% female	85.1% White	39.81 (4.76)	93.6% female	NR
Donovan and March (2014)^b^; BRAVE-ONLINE	23	3.96 (0.64)*	52.2% female*	NR (nationality only)	Mothers: 36.33 (4.83); fathers: 38.37 (6.28)*,**	Mostly mothers*,**	NR
Creswell et al. (2024); OSI	222	9.31 (1.83)	57% female	87% White	39 (5.93)	96% female	91% White
Green et al. (2023)^c^; OSI	40	8.88 (0.38)*	65% female*	79% White British	41.57 (3.83)*	90% female*	NR
Hill et al. (2022); OSI	23	9.65 (1.19)	73.9% female	65.2% White British	NR	95.6% mothers	NR
Poetar et al. (2024); ParentKIT	21	10.86 (2.43)	66.7% female	NR	40.43 (3.01)	81% female	NR
Dadds et al. (2019)^d^; AccessEI	66 / 35	6.79 (1.68)/7.49 (2.92)	22.4% female/20% female	NR	NR	46% both mother and father; 49% mother only* ^e^	NR
Enebrink et al. (2012); iComet	58	6.71 (2.31)	46.6% female	NR (nationality only)	NR	69.2% both mother and father; 27.9% mother only **	NR
Ghaderi et al. (2018); iComet	109	NR	NR	NR	NR	NR	NR
A. Sourander et al. (2016); SFSW	232	4	37.3% female	NR	Mothers: 30.5 (NR); fathers: 33.2 (NR)	NR	NR
A. Sourander et al. (2022); SFSW	600	4	39.7% female	NR	Mothers: 30.3 (NR); fathers: 32.7 (NR)	NR	NR
S. Sourander et al. (2022); SFSW	50	NR	26% female; 74% male	NR	Mothers: 31.9 (NR); fathers: 32.8 (NR)	NR	NR
Franke et al. (2020)^f^**; Triple-P Online	27	4 (NR)	71.7% male	NR (nationality only)	35.4 (4.87)	100% mothers but some (*n* = 43) fathers also took part	NR

*Data provided by author. **Data includes the control arm rather than just the intervention arm. *NR* not reported. *CD* conduct disorder. *ODD* oppositional defiant disorder. Digital intervention abbreviations: *ACE* Anxiety treatment for Children through online Education; *OSI* Online Support and Intervention for child anxiety; *SFSW* Strongest Families Smart Website. Diagnostic measure abbreviations: *SCAS-8* Spence Children's Anxiety Scale; *SDQ* Strengths and Difficulties Questionnaire; *ECBI* Eyberg Child Behavior Inventory; *DBD* Disruptive Behavior Disorders Rating Scale; *WWP* Werry–Weiss–Peters activity rating scale; *PACS* Parental Account of Child Symptoms

^a^Mazenc (2021): whilst 93 families started the intervention, demographics were only reported for the 47 completers. ^b^Donovan and March (2014) : although the mean age for the intervention arm was just under 4 years old, this study was included in review as the overall mean age was 4.08 years. The parent demographics relate to the whole sample. ^c^Green et al. (2023): 47 families received the intervention across both studies, however only the second study met the inclusion criteria for this review, which had 40 participants. Child ethnicity refers to the full sample rather than the 40 children. ^d^Dadds et al. (2019): the forward slash reflects Study 1/Study 2. Where no forward slash is used, the information was the same across both studies. ^e^Dadds et al. (2019): Parent gender was calculated by the review authors using data provided by the study author, and relates to the intervention groups of both studies combined. ^f^Franke et al. (2020): only reported demographic information for the whole sample, rather than just the intervention arm

Notably, age and gender differed according to whether the intervention targeted an emotional problem or a behavioural problem, in that studies where the intervention focused on an emotional problem featured a higher mean proportion of females (61.5%) and a higher average age (8.7 years old). Conversely, the mean age for the behavioural problem studies was lower—children were 5.5 years old on average, however this calculation excludes Ghaderi et al. (2018) and S. Sourander et al. (2022) who did not report mean ages, but included children in the age range of 10–13 years and 3–8 years, respectively. The mean percentage of female children was 31.5%.

Despite all included studies focusing on a parent-led intervention, there was a lack of basic parent demographic information provided, across both the emotional and behavioural focused studies. Parent age and gender were only reported in seven (54%) and eight (62%) studies respectively. Of those studies that did report it, the average parent age was 38.8 years (range: 36.3–41.6 years) for the emotional problem studies and 32.4 years (range: 30.5–35.4) for the behavioural problem studies. Five studies (all focused on emotional problems) provided a gender percentage for the primary parent; on average, 91% of the primary parents were females. In some of the behavioural problem studies, both parents were encouraged to take part in the intervention and three of these studies reported gender in terms of the percentage of children who had both parents participating, mother only participating or father only participating. Parent ethnicity was even less commonly reported, with only one study (Creswell et al., 2024) giving the ethnic background of parents (91% White).

Twelve studies (92%) reported at least some level of data regarding socio-economic status (SES), and most families had relatively high SES. Across all studies that reported parental education level, almost half of families (49%) had received a university education, broken down as 41.6% of families for the emotional problem studies and 52.7% for the behavioural problem studies. In terms of household income, this was reported in some form in six studies but was difficult to compare due to the different currencies used. One study (Ghaderi et al., 2018) used descriptive terms rather than monetary categories to classify income, with the majority of families describing their incomes as ‘sufficient—we don’t worry’. In studies which reported the percentage of families within particular income brackets, this was variable across each study, with some reporting that the families were in the highest income bracket (Donovan & March, 2014; Mazenc, 2021) and others having a fairly even spread across the various income brackets (Creswell et al., 2024; Dadds et al., 2019; Franke et al., 2020).

## Interventions

A total of eight different digital interventions were used across the 13 included studies. As can be seen in Table 4, two interventions, Online Support and Intervention for child anxiety (OSI; Creswell et al., 2024; Green et al., 2023; Hill et al., 2022) and the Strongest Families Smart Website (SFSW; A. Sourander et al., 2016, A. Sourander et al., 2022; S. Sourander et al., 2022), were the focus of three studies each, and iComet was used in two studies (Enebrink et al., 2012; Ghaderi et al., 2018), excluding the follow-up studies with no new participants. The remaining interventions were only the focus of one study: AccessEI (Dadds et al., 2019), ACE (Anxiety treatment for Children through online Education; Mazenc, 2021), BRAVE-ONLINE (Donovan & March, 2014), ParentKIT (Poetar et al., 2024) and Triple-P Online (Franke et al., 2020). Table 5 reports a summary of each included intervention.

**Table 5:** Intervention details

Intervention name; target problem	Theoretical basis	Intervention length for the parent (excluding guidance)	Full intervention duration for parent (excluding guidance)
ACE; anxiety	CBT	7 lessons (individual lesson duration NR), delivered over 9 weeks	NR
BRAVE-ONLINE; anxiety	CBT	8 modules (lasting around 60 min each), including two booster sessions, first 8 modules delivered weekly ^a^	10 h^b^
OSI; anxiety	CBT	8 modules (lasting around 30 min each) including a follow-up module, first 7 modules delivered weekly	4 h^b^
ParentKIT; anxiety & depressive symptoms	CBT	9 modules (lasting around 60 min each*, plus downloadable materials taking 30 min to read*), delivered over 3 weeks	13.5 h^b^
AccessEI; conduct	SLT, attachment theory, family systems	6 modules (lasting between 7 and 19 min each) delivered over 6–10 weeks	1 h 14 min
iComet; conduct	SLT, CBT	7 sessions (lasting around 90 min each), delivered over 10 weeks	10.5 h^b^
SFSW; disruptive behaviour	SLT, CBT, positive parenting	13 modules (lasting an average of 48 min each^c^) including two booster sessions, first 11 modules delivered weekly	10.4 h^b^
Triple P Online; ADHD	SLT, positive parenting	8 modules (lasting around 60 min each*), delivered over up to 16 weeks	8 h^b^

* Data provided by author. *NR* = not reported. *Digital intervention abbreviations: ACE* Anxiety treatment for Children through online Education; *OSI* Online Support and Intervention for child anxiety;* SFSW* Strongest Families Smart Website. *Theoretical abbreviations*:* CBT* Cognitive Behavioural Therapy; SLT: Social Learning Theory

^a^Intervention time period retrieved from Spence et al. (2008). ^b^Total duration of intervention not explicitly reported, but calculated by multiplying the number of modules by the average or approximate module length. ^c^Average module length reported in S. Sourander et al. (2022)

### Targeted Problems

In terms of the primary problem that the eight interventions aimed to address, there was an even split across EBP, with four interventions addressing emotional problems, and four addressing behavioural problems. All interventions for emotional problems focused at least partly on anxiety–-for three interventions (ACE, BRAVE-ONLINE and OSI), anxiety problems were the sole focus, whilst ParentKIT was a transdiagnostic intervention that targeted depressive symptoms in addition to anxiety symptoms. No interventions targeted a specific anxiety subtype—all addressed symptoms and interference relevant to all forms of anxiety problems. Out of the behavioural focused interventions, conduct problems were the most commonly targeted difficulty. Three interventions (SFSW, iComet and AccessEI) focused on conduct problems and/or disruptive behaviour, whilst Triple-P Online was aimed at children with ADHD.

### Intervention Durations

As seen in Table 5, studies used different terms to refer to the content completed by parents, including ‘modules’, ‘sessions’ and ‘lessons’, but ‘modules’ was the most commonly used term and this will be used from hereon. In terms of the self-directed aspects of the interventions (the core module content and activities that made up the intervention, and excluding guidance), there were inconsistencies in how studies reported the length of individual modules and the overall intervention length. For instance, some studies only reported how long it took the parents to complete the modules in weeks, with no details about the length of the individual modules, making it difficult to establish the overall amount of time parents took to complete the intervention.

For emotional problems, based on the available information, OSI was the briefest intervention in terms of the amount of time parents are recommended to spend going through the module material, with the eight modules each taking approximately 30 min to work through (giving a total time of approximately 4 h for parents to complete the module content). In contrast, for ParentKIT, parents complete nine modules containing about 60 min work for the parent, plus an additional 30 min per module to read downloadable material, over a three-week period (giving a total time of approximately 13.5 h for parents to complete the module content).

For behavioural problems, the reported duration for AccessEI was considerably shorter than any other intervention featured in this review. The six modules took between 7 and 19 min each for parents to work through, totalling 74 min for the full intervention. However, it should be noted that the online educational videos (which were part of the treatment) were not included in this figure, and therefore the actual intervention duration was longer. iComet required the most parent time, taking around 90 min for parents to complete each of the modules, bringing the total amount of time to 10.5 h.

### Guidance and Guidance Duration

The ‘guidance’ component of the interventions varied, with students (of all levels, from undergraduate to doctoral), psychologists, psychotherapists and research assistants providing support to parents across studies. An even wider range of facilitators were featured in a pragmatic RCT (Creswell et al., 2024) in which any practitioners who routinely delivered treatment for child anxiety problems could deliver OSI in the trial. In other studies, the intervention was delivered solely by students (BRAVE-ONLINE and ACE) or only by licenced health care professionals (SFSW). The modality in which the guidance was provided was most commonly via telephone, but emails, chat functions and video calls were also used. ACE and ParentKIT only provided support via written communication, and thus were the only interventions featured in this review not to provide any form of synchronous guidance. The content of the guidance provided was fairly similar across all interventions. As per the exclusion criteria for this review, guidance had to consist of more than just technical help—support, feedback or encouragement relating to the intervention content should be provided. The interventions used the facilitators to ‘check in’ with parents, to help them personalise the content to their child, to problem solve, and to check their understanding and implementation of the material. Table 6 provides an outline of the guidance provided for each of the eight interventions.

**Table 6:** Guidance provided by intervention facilitators

Intervention name; target problem	Facilitator profession	Facilitator contact time	Nature of guidance provided
ACE; anxiety	Graduate students	Provided weekly but otherwise NR	Weekly check-in messages, answering queries and providing support
BRAVE-ONLINE; anxiety	4th year psychology students	10 min after each module to type email; 15–30 telephone call part way through the intervention	Email contact after each module and one phone call part way through the intervention
OSI; anxiety	Various but mainly CWPs/EMHPs	20-min weekly telephone/video call	Weekly call to personalise content, overcome barriers, apply strategies and problem solve
ParentKIT; anxiety & depressive symptoms	Psychotherapists (trainee supervisors)	Ad hoc; time spent not recorded*	Communicating with parent using chat function, providing feedback and support and answering queries
AccessEI; conduct	Clinical psychologist or trainee clinical psychologist	50–60-min weekly video call	Reviewing parents' understanding and implementation plans
iComet; conduct	Research assistants, students and psychologists	Support provided through the platform; mean total time 310 min	Feedback and problem solving
SFSW; disruptive behaviour	Licensed health care professionals	35–45-min weekly telephone call	Weekly call to review application of new skills, answer questions and provide encouragement
Triple P Online; ADHD	Triple P facilitator	Two 20-min telephone consultations	Consultations to tailor the intervention and problem solve

*Data provided by author. *NR* not reported. *CWP* Children’s Wellbeing Practitioner. *EMHP* Education Mental Health Practitioner. *Digital intervention abbreviations: ACE* Anxiety treatment for Children through online Education; *OSI* Online Support and Intervention for child anxiety; *SFSW* Strongest Families Smart Website

For emotional problems, interventions typically provided at least some of the support via a written method, with ACE involving weekly check-in messages, BRAVE-ONLINE involving email contact after each module, and ParentKIT involving ad-hoc use of a chat function. Only two interventions reported the duration of the guidance (OSI and BRAVE-ONLINE), and for both of these interventions it was brief: parents receiving OSI had a 20-min telephone or video call after each module (totalling 160 min of support across the whole intervention), whilst BRAVE-ONLINE offered parents a single telephone call (lasting between 15 and 30 min) partway through the intervention. Coupled with the email support after each module (taking 10 min to type, totalling 100 min across the whole intervention, including booster sessions), this meant the total duration of guidance during BRAVE-ONLINE was 115–130 min. Guidance was generally provided routinely after each module for the emotional problems interventions, with the exception of ParentKIT.

For the behavioural interventions, AccessEI and SFSW provided weekly calls to families, either via telephone or video conferencing. Notably, AccessEI had the lengthiest guidance (of those that reported it) of all interventions featured in this review, with 50–60 min weekly calls (totalling 500–600 min of support over the intervention), perhaps to compensate for the relatively short modules for parents. This was closely followed by SFSW, in which the 35–45-min weekly calls totalled 455–585 min across the intervention. For iComet, all support was provided via the intervention platform, and Enebrink et al. (2012) reported a mean total support time of 310 min per research assistant for the duration of iComet in their study. Triple-P Online was considerably shorter in terms of the guidance duration, with only two telephone calls (lasting 20 min each) being offered during the intervention, giving a total support time of just 40 min.

### Intervention Functionalities

**Fig. 2:**
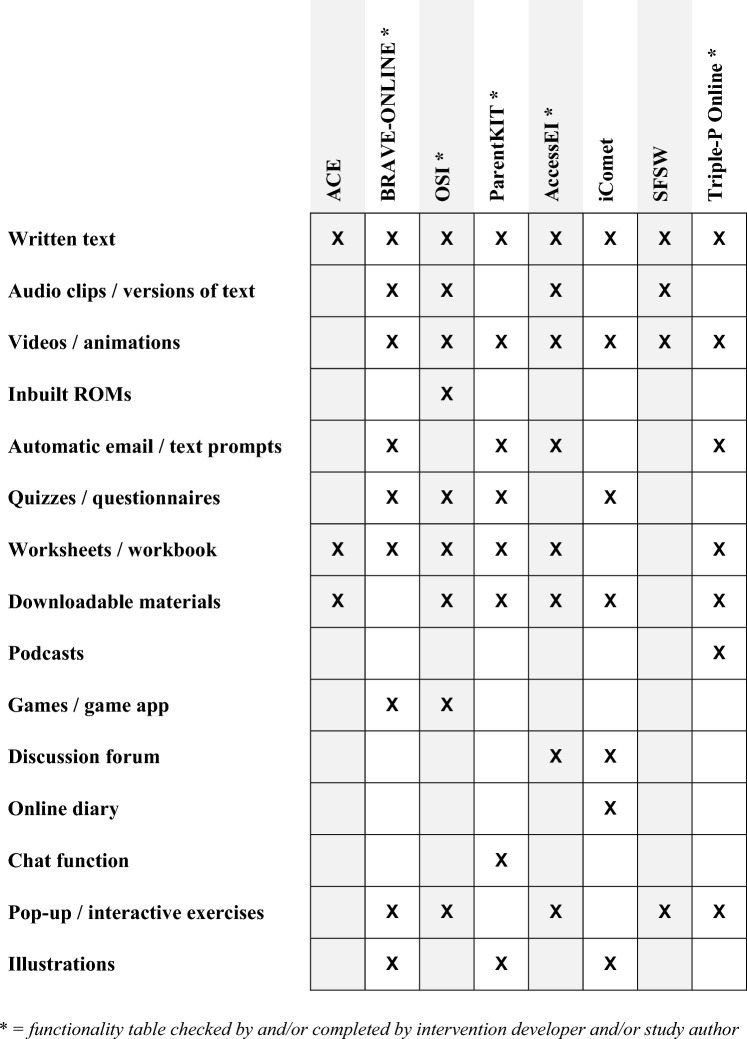
Intervention functionalities

Interventions were all website-based. No interventions were designed as mobile applications, and it was not stated in any of the studies whether the intervention was optimised for use on a smartphone. One intervention (OSI) did feature an optional game app for children (‘Monster’s Journey’) but this did not contain specific therapeutic content and was optional for families to use to help motivate their child if wanted, rather than being a core part of the intervention. Figure 2 illustrates the technical functionalities of each intervention.

Interventions were largely similar in terms of functionality, and there were no substantial differences between the interventions aimed at emotional problems and those aimed at behavioural problems. Many were of a modular format and involved reading text (100% of the interventions); watching videos or animations (87.5%); completing online worksheets or a workbook (75%); downloading materials (75%); and completing interactive exercises (62.5%). Some functions were unique to only one of the interventions included in this review; for example, inbuilt routine outcome measures (ROMs) were only featured in OSI, a chat function only featured in ParentKIT, an online diary only featured in iComet, and podcasts only featured Triple-P Online.

### Intervention Therapeutic Components

**Fig. 3:**
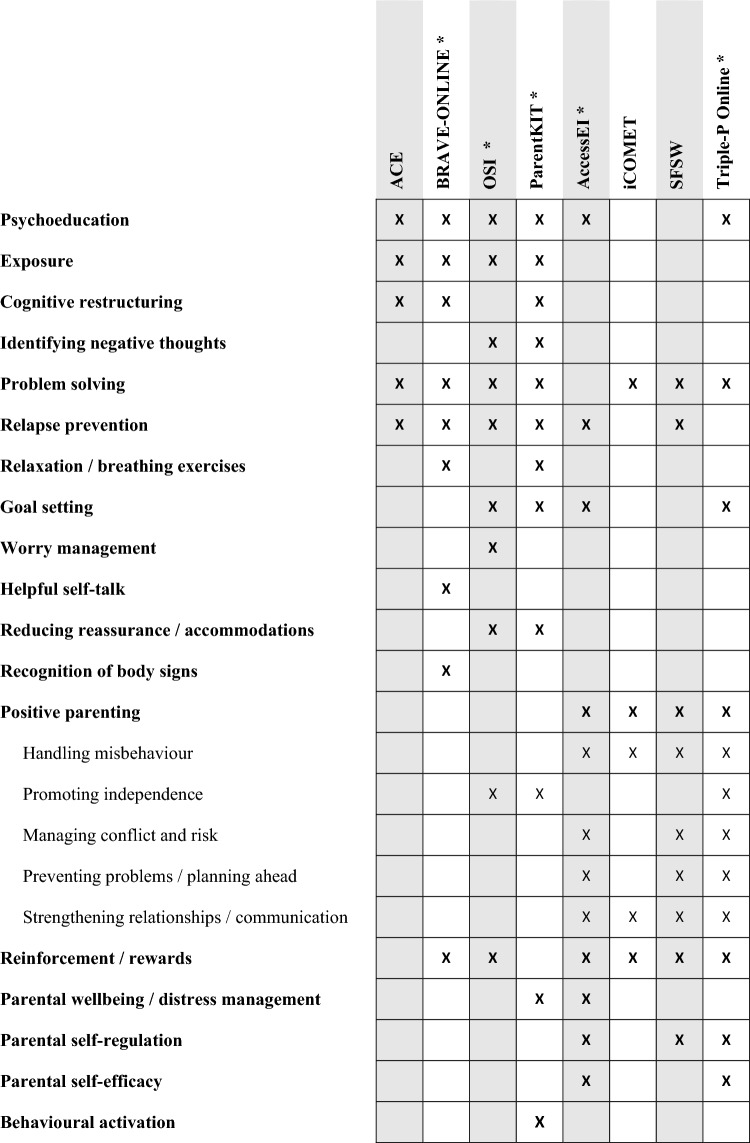
Intervention components

With regards to the therapeutic components of the interventions, all specified their theoretical basis, and for most this was at least partly CBT (75% of interventions). Considering this focus on CBT, it is not surprising that the therapeutic components of most interventions centred on common CBT concepts of problem solving (87.5% of the interventions), psychoeducation (75%) and exposure (50%). Positive parenting tended to be the key component tor interventions that targeted behavioural problems, with aspects of positive parenting featuring in all four interventions for behavioural problems. Figure 3 illustrates the therapeutic components of each intervention.

All four of the interventions for emotional problems identified CBT as their theoretical basis, with Poetar et al. (2024) further specifying that ParentKIT was based on Rational Emotive Behaviour Therapy (REBT). All four featured psychoeducation, exposure, problem solving and relapse prevention. OSI was the only intervention not to feature cognitive restructuring, but did involve identifying and testing negative thoughts (through action). OSI and ParentKIT were the only interventions to include goal setting, reducing reassurance/accommodations and promoting independence. BRAVE-ONLINE was unique in being the only intervention to feature helpful self-talk and recognition of body signs, whilst OSI was the only intervention to include worry management. As a transdiagnostic intervention that addressed both anxiety and depressive symptoms, ParentKIT was the only intervention to include behavioural activation. Parental wellbeing was also addressed in ParentKIT.

The interventions for behavioural problems had a mixed theoretical basis, but all four were based to some extent on social learning theory. Other theoretical bases included attachment theory (AccessEI), family systems (AccessEI) and CBT (iComet and SFSW). All interventions encompassed elements of positive parenting; specifically, all addressed handling misbehaviour, strengthening parent–child relationships/communication, and reinforcement/rewards. In 75% of these interventions, parents were taught skills for managing conflict and risk, preventing problems/planning ahead, and their own self-regulation. Several of the behavioural interventions also made use of typically-CBT components, with problem solving featuring in 75% of the interventions, and psychoeducation, relapse prevention and goal setting featuring in 50%.

## Outcomes

Completion rates, measures and outcomes are summarised in Table 7.

**Table 7:** Completion rates, primary outcome measures, timepoints and within-group effect sizes (Cohen’s *d*). Comparator and between-group effect sizes for RCTs (Cohen’s *d*)

First author (date); intervention	Completion rates / drop out	Primary outcome measure	Within-group effect sizes*	Between-group effect sizes, post tx*
Mazenc (2021); ACE	51.6% completed the full intervention	SCAS	Pre tx to post tx: *d* = 0.83^□^** Pre tx to 3 m FU: *d* = 1.04^□^**	N/A
Donovan and March (2014); BRAVE-ONLINE	73.9% completed Session 5 and 39.1% had completed all 6 sessions by the 6 m FU point	CGAS	Pre tx to post tx: *d* = 0.59^□^** Pre tx to 6 m FU: *d* = 1.66^□^**	Waitlist *d* = 0.67^□^**
Creswell et al. (2024); OSI	85.1% of those who started the intervention received at least 5 sessions	CAIS	Pre tx to 3 m post-rand: *d* = 0.46 ^∆^ ** Pre tx to 6 m post-rand: *d* = 0.58 ^∆^ **	Usual tx *d* = 0.01 ^∆^**
Green et al. (2023); OSI	65% completed Modules 0–4; 55% completed all modules^a^	CORS	Pre tx to post tx: *d* = 0.78 ^∆^ Pre tx to 1 m FU: *d* = 0.81 ^∆^	N/A
Hill et al. (2022); OSI	87% completed Modules 0–4	RCADS (total anxiety raw score)	Pre tx to post tx: *d* = 0.88^□^ Pre tx to 1 m FU: *d* = 1.1^□^	N/A
Poetar et al. (2024); ParentKIT	66.7% completed more than four modules; 38.9% completed all nine modules	SDQ (emotional problems subscale)	Pre tx to post tx: *d* = 0.85^∆^ Pre tx to FU: N/A	Waitlist *d* = 1.25^∆^**
Dadds et al. (2019); Study 1 AccessEI	16.4% dropout post tx, further 5.4% at FU; mean modules completed: 7.51	CRS (oppositional /defiant score)	Pre tx to post tx: *d* = 1.2^□^** Pre tx to 3 m FU: *d* = 1.01^□^**	Face-to-face intervention *d* = 0.24^□^**
Dadds et al. (2019); Study 2 AccessEI	5.7% dropout post tx, further 3% at FU; mean modules completed: 7.15	CRS (oppositional /defiant score)	Pre tx to post tx: *d* = 0.89^□^** Pre tx to 3 m FU: *d* = 0.63^□^**	Face-to-face intervention *d* = 0.28^□^**
Enebrink et al. (2012); iComet	65.5% completed all seven sessions; 46.6% completed FU	ECBI (intensity scale)	Pre tx to post tx: *d* = 1.23^□^** Pre tx to 6 m FU: *d* = 2.66^□^** Pre tx to 18 m FU: *d* = 2.9^b^^□^**	Waitlist *d* = 0.42^∆^/0.66^□^
Ghaderi et al. (2018); iComet	61% started the intervention	DBD (oppositional /defiant subscale)	Pre tx to post tx: *d* = 1.1^∆^ Pre tx to 1 yr FU: *d* = 0.53^∆^ ** Pre tx to 2 yr FU: *d* = 0.5^∆^ **	Family Check Up *d* = 0.23 ^∆^ **
A. Sourander et al. (2016); SFSW	24.1% discontinued the intervention; 75.9% completed ^c^	CBCL (externalising scale)	Pre tx to 6 m post-rand:: *d* = 0.76 ^∆^ Pre tx to 1 yr post-rand: *d* = 0.81 ^∆^ Pre tx to 2 yr FU: *d* = 0.9 ^d ∆^	Educational control *d* = 0.3 ^∆^ **
A. Sourander et al. (2022); SFSW	14.3% discontinued the intervention; 85.7% completed	CBCL (externalising scale)	Pre tx to 6 m after starting tx: *d* = 0.51 ^∆^ ** Pre tx to FU: *d* = N/A	RCT (A. Sourander et al., 2016) *d* = 0.08 ^∆^ **
S. Sourander et al. (2022); SFSW	88% completed the intervention	SDQ (total)	Pre tx to post tx: *d* = 1^∆^ ** Pre tx to 6 m FU: *d* = 1.16 ^∆^ ** Pre tx to 2 yr FU: *d* = 0.62 ^e ∆^ Pre tx to 2 yr FU: *d* = 0.47 ^e ∆^	N/A
Franke et al. (2020); Triple P Online	55% completed all eight modules	Conners EC-BEH (hyperactivity/inattention score)	Pre tx to post tx: *d* = 1.15 ^∆^ ** Pre tx to 6 m FU: *d* = 0.89 ^∆^	Waitlist *d* = 0.52 ^∆^

*The direction of the effect sizes have been standardised and indicate reductions in the child’s anxiety symptoms/interference levels. **For these timepoints, Cohen’s d was not reported in the paper. Effect sizes were calculated by the review authors using means and standard deviations (converted from standard errors when needed). □ Completer analysis. ∆ Intent-to-treat analysis. *NR* not reported. *Tx* treatment. *yr* years. *m* months. *Post-rand* post-randomisation. *FU* follow-up. *CD* conduct disorder. *ODD* oppositional defiant disorder. *Digital intervention abbreviations: ACE* Anxiety treatment for Children through online Education; *OSI* Online Support and Intervention for child anxiety; *SFSW* Strongest Families Smart Website. *Outcome measure abbreviations: SCAS* Spence Children's Anxiety Scale; *CGAS* Children's Global Assessment Scale; *CAIS* Child Anxiety Impact Scale; *CORS* Child Outcome Rating Scale; *RCADS* Revised Children's Anxiety and Depression Scale; *SDQ* Strengths and Difficulties Questionnaire; *CRS* Conners’ Rating Scale; *ECBI* Eyberg Child Behavior Inventory; *DBD* Disruptive Behavior Disorders Rating Scale; *CBCL* Child Behavior Checklist. *Conners EC-BEH* Conners Early Childhood Behavior scale

^a^Green et al. (2023): completion data only available for the whole sample, rather than the subset who screened positive for anxiety problems. ^b^Enebrink et al. (2012): 18 month follow-up reported in Högstrom et al. (2015). ^c^A. Sourander et al. (2016): completion rates NR in this paper but taken from A. Sourander et al. (2022). ^d^A. Sourander et al. (2016): 2 year follow-up reported in A. Sourander et al. (2018) ^e^S. Sourander et al. (2022): 2 year follow-up reported in S. Sourander et al. (2024)

### Completion Rates

All studies reported completion or dropout rates to some extent, although the heterogeneity in how it was reported complicates comparisons across studies. For instance, some studies (e.g. Mazenc, 2021) considered families to have ‘dropped out’ unless they completed every single module. Other studies, such as those featuring BRAVE-ONLINE and OSI, identified a point in the intervention where the key therapeutic content had been completed (for both these interventions, this was the fifth module), and reported completion rates in relation to that. One study (Dadds et al., 2019) only reported attrition in relation to the study outcome measures, rather than the intervention itself.

Few studies outlined reasons why families dropped out of the intervention (as opposed to not completing the study measures), but those that did reported reasons such as technical difficulties with the digital intervention, the child’s anxiety resolving, the family disengaging from the service (Hill et al., 2022), the family accessing another service, or parents being unable to attend sessions (Dadds et al., 2019). Ghaderi et al. (2018) noted that a larger number of participants dropped out following randomisation to iComet compared to the control intervention, and hypothesised that this may be to do with treatment expectations.

Of the interventions for emotional problems, the largest number of dropouts were reported for ACE (Mazenc, 2021), with only just over 51% of families completing the intervention. They explored predictors of completion, and identified that parents with a higher level of education, who had lower levels of depression, anxiety and stress themselves, and whose child had higher anxiety symptoms at baseline, were more likely to complete ACE. In contrast, BRAVE-ONLINE and OSI, as mentioned above, consider completion of the first five modules to be adequate as a ‘dose’ of the intervention, and completion of this module ranged from 65% (Green et al., 2023) to 87% (Hill et al., 2022) for OSI, and was 73.9% for BRAVE-ONLINE (Donovan & March, 2014). Poetar et al. (2024) reported completion rates of 66.7% for more than four modules of ParentKIT. When using the same metric as Mazenc (2021) when considering who is classed as a ‘completer’ (i.e. completion of all modules) across interventions, completers ranged from 38.9% (for ParentKIT; timing by which the intervention had to be completed not specified), closely followed by 39.1% (at the 6 month post-treatment assessment for BRAVE-ONLINE), to 55% for OSI during the duration of the study conducted by Green et al. (2023).

Of the interventions for behavioural problems, Triple-P Online (Franke et al., 2020) had comparable completion rates (following Mazenc’s (2021) definition) to the anxiety-focused interventions with 55%. For iComet, Ghaderi et al. (2018) only reported the proportion of families who started the intervention (61%) rather than any data regarding completion. However, Enebrink et al. (2012) reported 65.5% completion of all seven iComet sessions. Dadds et al. (2019) reported low dropout rates; however, this was in relation to completion of the study questionnaires, rather than the intervention, with 16.4% of participants not completing the post-treatment measures in their first study, and only 5.7% in their second study. That said, they did report that families in the intervention arm received a significantly higher treatment dosage than those who received the face-to-face control intervention (in terms of number of sessions completed and the amount of time spent in treatment) in their first study, but this difference was not significantly higher in their second study.

### Primary Outcome Measures and Follow-Up Periods

Given the range of EBP addressed in this review, it is unsurprising that a variety of different primary outcome measures were used, although notably this was even the case across studies using the same intervention. A total of twelve different primary outcome measures were used across the thirteen included studies, and these were almost all parent-report. Donovan and March (2014) were the exception in using a clinician-report measure as the primary outcome. In line with best practice for RCTs some studies collected primary outcomes measures in relation to randomisation date, whereas others collected them according to dates of intervention administration. Follow-up periods ranged from 1 month from the end of completion of core treatment modules (Green et al., 2023; Hill et al., 2022) to 2 years (Ghaderi et al., 2018). Three studies (Enebrink et al., 2012; A. Sourander et al., 2016; S. Sourander et al., 2022) reported further follow-up data in additional papers. Only one study (Poetar et al., 2024) did not report any follow-up measures, and instead reported data from just two timepoints. Two studies (Donovan & March, 2014; Enebrink et al., 2012) only reported on follow-up measures for the intervention group.

For the studies focused on emotional problems, three studies used measures of symptoms as the outcome that we have identified as being the primary measure, whilst three (Creswell et al., 2024; Donovan & March, 2014; Green et al., 2023) used measures of interference/functioning. The follow-up periods used in the emotional problem studies were relatively short, with the longest follow-up periods being only 6 months (Creswell et al., 2024; Donovan & March, 2014), and the average follow-up period being 3.4 months.

The studies focused on behavioural problems had this focus for their primary outcome, often focusing on a particular subscale of a measure (e.g. the oppositional/defiant score of the Conners’ Rating Scale (Dadds et al., 2019); the intensity scale of the Eyberg Child Behavior Inventory (Enebrink et al., 2012)). Compared to the emotional problem studies, follow-up periods were typically longer (the average follow-up period was 9.5 months), with the longest follow-up period being 2 years. Additional follow-up periods were reported in later papers for three studies: an 18-month follow-up for Enebrink et al. (2012), reported in Högström et al. (2015), a 2-year follow-up for A. Sourander et al. (2016), reported in A. Sourander et al. (2018), and a 2-year follow-up for S. Sourander et al. (2022), reported in S. Sourander et al. (2024).

### Study Outcomes

All studies reported meaningful improvements following treatment for the intervention group, with treatment effects superior to waitlist controls or either comparable to or superior to active controls (for the RCTs). Effect sizes (Cohen’s *d*) are reported in Table 7. Across all studies, within-group effect sizes ranged from ranged from small to large immediately post-treatment for the intervention groups, and were at least moderate by follow-up.

The largest within-group effect sizes for the emotional problem interventions, as calculated using available data for the primary outcome measure, were for BRAVE-ONLINE (Donovan & March, 2014), with a large effect size (*d* = 1.66) at the 6 month follow-up timepoint for the completer sample. Donovan and March (2014) also reported that over 70% of families who fully completed BRAVE-ONLINE were free of their primary anxiety diagnosis by the 6 month follow-up. For OSI, large effect sizes were reported by Green et al. (2023) and (Hill et al., 2022), and were in the moderate range in Creswell et al. (2024), although notably this was based on intent-to-treat analyses in a real-world setting where over a fifth of participants had not started treatment by the end of the trial. For the remaining studies focusing on emotional problems, effect sizes were all large for reductions in children’s anxiety symptoms (Mazenc, 2021) and internalising problems (Poetar et al., 2024).

Three of the emotional problem interventions were evaluated as part of an RCT. Two of these interventions were compared to a waitlist control group, and resulted in medium (BRAVE-ONLINE; Donovan & March, 2014) and large (ParentKIT; Poetar et al., 2024) between-group effects immediately post-treatment for the primary outcome measure. Whilst the focus of this review was on continuous measures of symptoms/interference, it should be noted that the percentage of children free of their primary diagnosis post-treatment in Donovan and March (2014) was relatively low at 39.1%, although this rose over the 6-month follow-up (52.2% in the intent-to-treat sample; 70.6% in the completer sample). The third intervention, OSI, was the only intervention aimed at an emotional problem to be compared against an active comparator treatment. OSI was compared to the usual treatment that the participating services offered to children with anxiety problems (which was predominantly CBT). The study found clear evidence of non-inferiority, with negligible between-group differences, despite OSI taking significantly less time to deliver than usual treatment. OSI was also found to be likely to be a more cost-effective treatment than the usual treatment.

In terms of the interventions focused on behavioural problems, iComet had the largest within-group effect sizes immediately post-treatment (Enebrink et al., 2012; Ghaderi et al., 2018). Large effect sizes were also seen in Dadds et al. (2019), Franke et al. (2020), A. Sourander (2016) and S. Sourander (2022), with A. Sourander (2022) showing a medium effect size.

Four of the behavioural problem interventions were evaluated in RCTs. Triple-P was compared to a waitlist control (Franke et al., 2020) with moderate between-group effects immediately post-treatment. iComet was also compared to a waitlist control group (Enebrink et al., 2012) and similarly resulted in statistically significant small-moderate between-group effects favouring iComet, and notably did not differ significantly from an established parent training programme (Family Check Up; FCU) in Ghaderi et al. (2018). The two RCTs within Dadds et al. (2019) compared AccessEI to a face-to-face version of the intervention and both found very small and non-significant between-group effects immediately post-treatment. As such it was concluded that AccessEI could make treatment for conduct problems more accessible, particularly for families living rurally. A. Sourander et al. (2016) compared SFSW to an ‘educational control’ (EC) and found small between-group effects immediately post-treatment, significantly favouring SFSW. The EC involved parents accessing a website that introduced them to positive parenting strategies, supplemented with a 45-min call from a ‘coach’ who provided positive parenting advice. Whilst not an RCT, A. Sourander et al. (2022) compared the intervention group from A. Sourander et al. (2016) to participants receiving SFSW in routine practice, and comparisons between two groups revealed a negligible between-group effect size.

## Quality Assessment

Applying the QualSyst tool (Kmet et al., 2004) to the thirteen studies revealed that all the included studies were rated as being of good quality, and above the 75% mark that Kmet et al. (2004) suggest as a conservative cut-off point for article inclusion in a systematic review. In particular, we considered all studies to have used appropriate measures of assessment and to have drawn appropriate conclusions from their results. Studies tended to be marked down for lack of participant demographics (particularly concerning the parents, as highlighted earlier); not reporting an estimate of variance in the main results; not reporting a power analysis; only conducting/reporting completer analyses (as opposed to intent-to-treat); and for a lack of clarity regarding their randomisation procedures and the blinding of the study investigators.

**Fig. 4:**
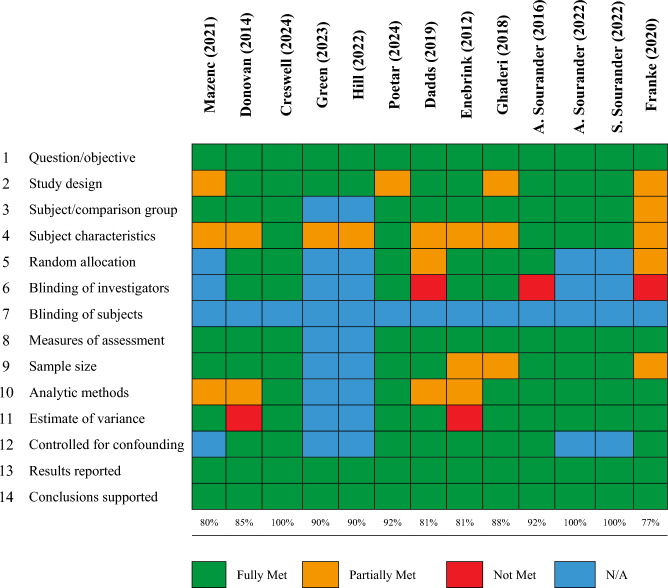
Quality ratings

Figure 4 illustrates the rating for each of the 14 items on the QualSyst tool for the thirteen studies, along with their percentage score. Appendix 9 lists each of the 14 items in full, along with the criteria used to allocate scoring and examples of how they were applied to the included studies in this review. Considering the nature of the interventions included in the studies, we did not deem it possible for any of the participants to be blinded, and therefore marked Item 7 as ‘not applicable’ for all studies. Items 5, 6, 7 and 12 were marked as ‘not applicable’ for the non-randomised studies, in accordance with the QualSyst guidelines. As Green et al. (2023) and Hill et al. (2022) explicitly identified themselves as case series, they were marked as ‘not applicable’ for Item 3, 5, 6, 7, 8, 9, 10, 11 and 12, again in accordance with the guidance.

There were no clear differences between the quality of the studies focusing on emotional problems compared to those focused on behavioural problems. The emotional problem studies had an average percentage score of 89% (range: 80–100%), whilst the average percentage score was 88% for the behavioural problem studies (range: 77–100%).

## Discussion

### Summary of Findings

Guided, parent-led digital interventions are a potential method of increasing access to treatment for preadolescent children with EBP, but there has been no synthesis of the evidence base for this particular type of intervention, nor the characteristics of the interventions. To address this gap, our review synthesised the findings of 13 studies covering eight interventions, half of which were aimed at parents of children with emotional problems (anxiety problems, anxiety and depressive symptoms) and the other half for parents of children with behavioural problems (ADHD, conduct problems and disruptive behaviour problems).

The primary aim of our systematic review was to establish the current evidence base of guided, parent-led digital interventions for children with EBP. The review demonstrated that there are a range of interventions available to potentially met the needs of this population and all studies reported a meaningful improvement in the child’s symptoms or interference levels after receiving the digital intervention, and within-group effect sizes (Cohen’s *d*) averaged at 0.87 (range: 0.36–1.22) immediately post-treatment. Studies were generally of very good quality, according to the QualSyst checklist (Kmet et al., 2004). On the whole, the favourable outcomes across interventions, coupled with the quality of the studies, suggests there is a promising evidence base for guided, parent-led digital interventions for children with EBP.

Eight of the included studies (62%) were RCTs, with half comparing the digital intervention to a waitlist and the other half to an active treatment. In the four RCTs with waitlist comparators, between-group effect sizes immediately post-treatment were moderate to large. For the studies with active comparators, between-group effects were negligible to small but did not require families to travel and were typically shorter in length, offering potential wider advantages in terms of increasing accessing and cost-effectiveness. However, only one study (Creswell et al., 2024) included a health economics analysis (with promising results), and further work is needed to clarify whether the other interventions are also better value for money than their face-to-face equivalents to inform decision making from healthcare providers and policymakers (Owen et al., 2018).

As well as outcomes, other aspects of the interventions need to be considered when assessing the strength of the evidence base. It was notable that some of the digital interventions suffered from substantial dropout, relative to non-digital treatments. Where reported, completion of the full intervention for emotional problems averaged at 46% (range: 39–55%), compared to, for example, reports of 83% completion for family-focused face-to-face treatments for anxiety problems (In-Albon & Schneider, 2006). Interventions for behavioural problems fared better overall, with completion of the full intervention averaging at 70% (range: 55–88%), higher than 49% completion reported in a review of face-to-face behavioural interventions (Chacko et al., 2016).

Considering that higher levels of engagement and attendance in treatment have been found to be associated with better outcomes (Nock & Ferriter, 2005), and that several studies only reported results for completer samples, this may have skewed the positive outcomes reported (Andrade, 2022). Not only does this lower confidence in the evidence base for these interventions, it also highlights the need to explore more about why families drop out of treatment, in order to ensure that treatment accessibility and outcomes are maximised, particularly for some of the interventions aimed at emotional problems.

It is important to note that all thirteen studies were conducted in developed, high income countries, and there is no evidence on how the included interventions would fare in countries with less resources and potential lower digital device use and internet capability. There was also a lack of ethnic variation—when reported, the majority of children were described as ‘white’. Likewise, families tended to be of relatively high SES, in terms of parental education and household income. These factors limit the generalisability of the evidence base to settings other than those featured in the included studies.

The second aim of this systematic review was to explore the characteristics of the included interventions. Considering the cost of developing new digital interventions, being aware of the components and functions of existing (good) interventions can provide a solid basis for the development and improvement of others (O'Cathain et al., 2019). Across the interventions, a variety of therapeutic components were applied, with CBT techniques (such as problem solving, exposure and cognitive restructuring) and positive parenting being the most commonly featured components in the emotional problems and the behavioural problems interventions respectively. With regards to technical functions, again a wide variety were used to enhance interactivity and engagement for parents, including more traditional therapeutic functions such as written texts and worksheets, as well as videos, quizzes, automatic prompts and pop-up exercises.

Interestingly, despite the rapid growth in the popularity of smartphone apps, particularly in the area of mental health (Torous & Roberts, 2017), no eligible app-based interventions were identified and studies did not comment on whether their digital interventions were optimised for use on a mobile device. Given the burgeoning use of mobile devices such as smartphones (rather than laptops or computers), particularly in low resource settings, improving the experience of those using the interventions via mobile devices may well further increase the accessibility of digital interventions (Price et al., 2014).

Along with the components and functions of the interventions, when developing new interventions for this population, it is useful to consider other elements, such as intervention length and the type of guidance offered. This varied substantially across the included interventions, with some only providing guidance once during the course of treatment, and others providing it after every module, or throughout treatment on an ad-hoc basis. In terms of the actual time this took facilitators in total over the course of the intervention, Triple-P Online had the shortest total recommended guidance duration of only 40 min, compared with AccessEI, where guidance could last up to 600 min. Generally, these durations represent a large time saving compared to traditional face-to-face treatments, bringing potential to increase treatment accessibility. One exception was AccessEI, in which, whilst there may have been accessibility benefits from the remote approach, the total overall treatment time was significantly greater than the face-to-face comparator treatment. Furthermore, several of the interventions did not require the guidance to be provided by a highly qualified or trained professional, with students, research assistants and trainees frequently providing support to parents, again bringing potential to increase accessibility by not requiring highly specialised therapists who are not widely available in many settings.

## Strengths and Limitations of the Included Studies

The studies were all of good quality overall. Each particular EBP featured in the review (anxiety problems; anxiety and depressive symptoms; ADHD; conduct problems; disruptive behaviour) had an intervention that is backed by at least one RCT, and almost all of the specific interventions (the exception being ACE) had been evaluated in an RCT. Whilst the quality of the RCTs was generally strong, aspects such as reporting on randomisation, blinding and power calculations were lacking in some studies. For example, some made no mention of the randomisation procedure or whether investigators were blind, and several studies did not reach their recruitment target and were therefore under powered (or did not report a power calculation at all). The exact timing of assessments was also often unclear (e.g. whether assessments were a certain number of weeks post-randomisation or from the end of treatment). Further high quality, fully powered trials are clearly needed to ensure confidence in many of the identified interventions.

In addition to being evaluated in RCTs in research settings, it was encouraging to see that some of the interventions (OSI, iComet and SFSW) had been tested in ‘real world’ environments. This is important to ensure that interventions that have been shown to be effective in controlled settings continue to work well for families in routine practice, as this is not always the case (e.g. Ginsburg et al., 2020). The pragmatic RCTs conducted by Creswell et al. (2024) and Ghaderi et al. (2018) both produced outcomes comparable to when the same interventions were tested in more research-based settings. Moreover, attempts to implement an intervention in routine practice following an RCT were noted to be successful by A. Sourander et al. (2022) —with very low dropout in the implementation study of SFSW, which the authors attributed to the intervention’s growing research base.

Many of the studies used parent-reported measures, which although appropriate and recommended for the preadolescent age range (Creswell et al., 2021), nonetheless have their limitations. For instance, differing agreement levels have been found between parent- and child-report questionnaires (Poulain et al., 2020). Whilst this review focused on the primary outcome measure of each study, it should be noted that some studies did include ratings of children and clinicians in their secondary outcomes, which help to provide a fuller picture of the intervention outcomes from different perspectives (Fuggle, 2015; Terrelonge & Fugard, 2017). Although all the interventions in this review produced positive outcomes in the short-term, for many of them we do not know if these effects were sustained, weakening confidence in the evidence base for these interventions, particularly for studies of interventions for emotional problems where the longest follow-up period was 6 months, in contrast to studies looking at behavioural problems which collected follow-up data up to 2 years post-intervention. Establishing the long-term outcome of these interventions will be important, given the somewhat mixed evidence more widely for the sustained effects of interventions across emotional problems (e.g. Ginsburg et al., 2014) and behavioural problems (e.g. Van Aar et al., 2017) in children and young people.

Studies often failed to report clear details concerning the proportion of participants who completed each module, and some did not report such information at all, instead only reporting retention to the study measures. As noted by MacDonell and Prinz (2017), inconsistencies with regards to how completion rates are reported across studies makes it difficult to gain a full picture of adherence to the interventions, however it did appear that drop out from treatment was an issue for many of the interventions, particularly those addressing anxiety problems.

Finally, many of the studies were co-authored by at least one developer of the intervention, which may have resulted in researcher bias (Eisner, 2009). For example, in wider fields reviews of the evidence have concluded that effect sizes from interventions are notably larger when programme developers are involved in the study than when they are conducted independently. Whilst there may be a number of reasons for this, for example relating to the fidelity of the delivery of the intervention, it is possible that these studies are subject to systematic bias (Eisner, 2009) and, as such, further independent evaluation will be valuable going forwards for the interventions included in this review.

## Strengths and Limitations of this Review

We conducted a comprehensive search using broad search terms, which was also supplemented with hand searching. We had no restrictions on date, publication status or study design, to allow a variety of studies to be included, and as a result this review included studies that have not appeared in previous systematic reviews. Efforts were made to contact the authors of all included studies, to gain missing information and to confirm the accuracy of the components and functionality coding system applied to the interventions. However, as only 53.8% of the authors were contactable, this left three interventions where the coding system relied solely on the description of the intervention in the paper. This was problematic given that research papers (including many of those in this review) typically only provide brief descriptions of the intervention, and reflects a wider issue of treatment manuals not being freely available (Cuijpers et al., 2024). Moreover, we did not seek verification on other details of the interventions, such as the guidance provided or the overall length, so conclusions drawn about these aspects were also reliant on the information provided in the papers. Since not all studies gave full details of the interventions and guidance, this limits the accuracy and thoroughness of the information in this review.

Despite the comprehensive search, we did not have robust translation facilities available and therefore were only able to include studies written in English. This may have resulted in studies that otherwise met the eligibility criteria being missed (Walpole, 2019). Also concerning the screening, it should be noted that Cohen’s kappa was only ‘fair’ (*k* = 0.26) between the two screeners at the title/abstract stage. However, this is likely due to the ‘Kappa Paradox’, in which a high number of agreements between reviewers (in this case of this review, high agreement in the number of articles excluded) causes a symmetrical imbalance, thereby resulting in a low kappa coefficient (Dettori & Norvell, 2020; Feinstein & Cicchetti, 1990). Percentage agreement however was 84%, above what is typically considered acceptable percentage agreement for a systematic review (Belur et al., 2021).

We were interested in a specific type of intervention and our eligibility criteria was therefore very tight; for example, the intervention had to be fully digital and fully led by the parent. This contrasts with previous reviews which required the intervention to only be a minimum of 50% online (e.g. Thongseiratch et al., 2020), included interventions with an equal number of parent sessions and child sessions (e.g. Grajdan et al., 2024), or focused on parent-led interventions amongst youth and family-focused interventions (e.g. MacDonell & Prinz, 2017). Whilst this meant our review is an accurate reflection of the type of intervention we set out to synthesise, it does mean that studies that may have closely met the criteria were excluded. For example, Aspvall et al. (2018) evaluated a therapist guided, digital intervention for preadolescents with obsessive compulsive disorder, but it was not included in the study due to the equal number of child and parent sessions. Such studies may have offered valuable insight to the range of functionalities and components that were identified, and allowed a wider variety of EBP to be represented in this review.

A wide range of functionalities and components were captured and coded in this review, and whilst these may help to form the basis of future interventions for the populations focused on in this review (O'Cathain et al., 2019), we were unable to draw conclusions about the impact of particular components on treatment outcomes due to the assortment of other potential moderators (such as intervention length, therapeutic content, etc.). This is important to bear in mind for the development of future interventions, as we do not know which components are critical for good treatment outcomes and parental engagement. Similarly, the nature of facilitator guidance differed between interventions, and coupled with these other potential moderators, meant we were unable to confidently establish their impact and draw conclusions about, for instance, the optimal format and length of guidance that parents require or prefer.

Another aspect that we were unable to draw conclusions about was parental engagement with the interventions. We did not focus our review on this area for a number of reasons, including the lack of engagement data typically reported in papers (Chacko et al., 2016) and the fact that there are recent reviews investigating the nature and extent of parental engagement in treatment for both emotional problems (e.g. Grajdan et al., 2024; Klein et al., 2024), and behavioural problems (e.g. Gonzalez et al., 2023; Sanchez et al., 2024). Nonetheless, enhancing parental engagement has been highlighted as critical and potentially links to improved outcomes (Haine-Schlagel & Walsh, 2015), and therefore a closer look at this association in future reviews would be helpful.

Not all papers reported effect sizes and given the available data in papers, there were instances where a between-groups effect size calculator had to be used instead of a within-groups effect size calculator (following e.g. Chessell et al., 2021; Halldorsson et al., 2021). The effect sizes for these studies should therefore be treated with caution, as between-groups effect size calculators assume statistical independence between intervention scores. We also commented on the quality of the studies, which contributed to our assessment of the evidence base, but several of the studies were case studies or other non-randomised designs, and therefore over half of the criterion in the quality checklist were not applicable (Kmet et al., 2004).

Finally, it should be acknowledged that three of the authors of this review have been involved in developing and/or evaluating one of the interventions featured (OSI), which may have resulted in bias. To mitigate this, one member of the review team was included who had not been involved in any previous OSI research and this team member was the second rater for title/abstract and full text screening, data extraction cross checking, and the quality assessment.

## Future Directions

Future studies evaluating guided, parent-led digital intervention for children with EBP should include RCTs that clearly report the randomisation process, who in the research team was blinded, and a power analysis in order to further increase confidence in the evidence base for these interventions. More generally, studies should collect and report key parent demographics, especially age and gender, so the impact of such demographics on intervention outcomes, delivery and acceptance can be evaluated. The long-term efficacy of these interventions also needs to be established. In addition, clear reporting of adherence to the intervention, such as the percentage of families who completed each module, is critical, as is improving diversity in the ethnicities and socio-economic status of the participating families and exploring the outcomes of such digital interventions in low- and middle-income countries.

Considering the positive outcomes reported by all studies included in this review, digital interventions, led by the child’s parent and with some guidance can be considered to be a potentially valuable method of increasing access to treatment for preadolescents with EBP. Anxiety problems and conduct problems were well addressed in this review, but we did not locate guided, parent-led digital interventions for, e.g. children with obsessive compulsive disorder or low mood, that met the review eligibility criteria, or interventions for specific anxiety disorders (such as social anxiety disorder or selective mutism). It will be important to establish the effectiveness of these broader interventions for more specific psychological difficulties, to identify where more specifically targeted platforms may be useful.

## Conclusions

Whilst more robust RCTs and implementation studies are required, overall, guided, parent-led digital intervention appears to be a potential solution to increasing access to treatment for families of children with EBP, but the lack of long-term follow-up data and variable dropout rates needs to be addressed. This review has given an overview of the functions, components and types of guidance to inform the development and improvement of guided, parent-led digital interventions, but future research is needed to identify which elements are associated with better outcomes and engagement.

## Supplementary Information

Below is the link to the electronic supplementary material.

Supplementary file1 (PDF 422 KB)

## Data Availability

No datasets were generated or analyzed during the current study.
