# Supplementary material for: A Systematic Review of Guided, Parent-Led Digital Interventions for Preadolescent Children with Emotional and Behavioural Problems
Source: Clin Child Fam Psychol Rev. 2025 May 11;28(2):414–38. doi: 10.1007/s10567-025-00521-x (PMC12162774; doi:10.1007/s10567-025-00521-x)
Supplement: Supplementary file 1 — Supplementary file1 (PDF 422 KB) [file 10567_2025_521_MOESM1_ESM.pdf]

Supplementary Information: Appendices 1-9

A systematic review of guided, parent-led digital interventions for preadolescent children with emotional and behavioural problems

Clinical Child and Family Psychology Review

Emily Whitaker, Chloe Chessell, Maxwell Klapow, Cathy Creswell

Corresponding author: Emily Whitaker

Departments of Experimental Psychology and Psychiatry,

University of Oxford, Oxford, UK

Contents

PRISMA Checklist (Appendix 1) 3

Synthesis Without Meta-analysis (SWiM) Reporting Items (Appendix 2) 6

Changes to Protocol (Appendix 3) 8

Search Strategy (Appendix 4) 10

Eligibility Criteria (Appendix 5) 15

Inclusion criteria 15

Exclusion criteria 16

Full Text Stepped Exclusion List (Appendix 6) 17

Full Text Exclusion Reasons (Appendix 7) 18

Examples of excluded studies that may appear to meet the eligibility criteria 22

Sample Characteristics – Further Details (Appendix 8) 23

Quality Assessment (Appendix 9) 27

PRISMA Checklist (Appendix 1)

Section and Topic	Item #	Checklist item	Location where item is reported
**TITLE**			
Title	1	Identify the report as a systematic review.	1
**ABSTRACT**			
Abstract	2	See the PRISMA 2020 for Abstracts checklist.	3
**INTRODUCTION**			
Rationale	3	Describe the rationale for the review in the context of existing knowledge.	6
Objectives	4	Provide an explicit statement of the objective(s) or question(s) the review addresses.	7
**METHODS**			
Eligibility criteria	5	Specify the inclusion and exclusion criteria for the review and how studies were grouped for the syntheses.	8-10; Appendix 5
Information sources	6	Specify all databases, registers, websites, organisations, reference lists and other sources searched or consulted to identify studies. Specify the date when each source was last searched or consulted.	7-8
Search strategy	7	Present the full search strategies for all databases, registers and websites, including any filters and limits used.	Appendix 4
Selection process	8	Specify the methods used to decide whether a study met the inclusion criteria of the review, including how many reviewers screened each record and each report retrieved, whether they worked independently, and if applicable, details of automation tools used in the process.	10
Data collection process	9	Specify the methods used to collect data from reports, including how many reviewers collected data from each report, whether they worked independently, any processes for obtaining or confirming data from study investigators, and if applicable, details of automation tools used in the process.	11-12
Data items	10a	List and define all outcomes for which data were sought. Specify whether all results that were compatible with each outcome domain in each study were sought (e.g. for all measures, time points, analyses), and if not, the methods used to decide which results to collect.	11-12; Tables 3-7
	10b	List and define all other variables for which data were sought (e.g. participant and intervention characteristics, funding sources). Describe any assumptions made about any missing or unclear information.	11-12; Tables 3-7
Study risk of bias assessment	11	Specify the methods used to assess risk of bias in the included studies, including details of the tool(s) used, how many reviewers assessed each study and whether they worked independently, and if applicable, details of automation tools used in the process.	12

Section and Topic	Item #	Checklist item	Location where item is reported
Effect measures	12	Specify for each outcome the effect measure(s) (e.g. risk ratio, mean difference) used in the synthesis or presentation of results.	11-12
Synthesis methods	13a	Describe the processes used to decide which studies were eligible for each synthesis (e.g. tabulating the study intervention characteristics and comparing against the planned groups for each synthesis (item #5)).	12-13
	13b	Describe any methods required to prepare the data for presentation or synthesis, such as handling of missing summary statistics, or data conversions.	11-12
	13c	Describe any methods used to tabulate or visually display results of individual studies and syntheses.	13
	13d	Describe any methods used to synthesize results and provide a rationale for the choice(s). If meta-analysis was performed, describe the model(s), method(s) to identify the presence and extent of statistical heterogeneity, and software package(s) used.	12-13
	13e	Describe any methods used to explore possible causes of heterogeneity among study results (e.g. subgroup analysis, meta-regression).	13
	13f	Describe any sensitivity analyses conducted to assess robustness of the synthesized results.	N/A
Reporting bias assessment	14	Describe any methods used to assess risk of bias due to missing results in a synthesis (arising from reporting biases).	12; Appendix 9
Certainty assessment	15	Describe any methods used to assess certainty (or confidence) in the body of evidence for an outcome.	N/A
**RESULTS**			
Study selection	16a	Describe the results of the search and selection process, from the number of records identified in the search to the number of studies included in the review, ideally using a flow diagram.	13-15
	16b	Cite studies that might appear to meet the inclusion criteria, but which were excluded, and explain why they were excluded.	Appendix 7
Study characteristics	17	Cite each included study and present its characteristics.	16; Table 3
Risk of bias in studies	18	Present assessments of risk of bias for each included study.	40-42
Results of individual studies	19	For all outcomes, present, for each study: (a) summary statistics for each group (where appropriate) and (b) an effect estimate and its precision (e.g. confidence/credible interval), ideally using structured tables or plots.	Table 7
Results of	20a	For each synthesis, briefly summarise the characteristics and risk of bias among contributing studies.	16-40

Section and Topic	Item #	Checklist item	Location where item is reported
syntheses	20b	Present results of all statistical syntheses conducted. If meta-analysis was done, present for each the summary estimate and its precision (e.g. confidence/credible interval) and measures of statistical heterogeneity. If comparing groups, describe the direction of the effect.	Table 7
	20c	Present results of all investigations of possible causes of heterogeneity among study results.	Tables 3-7
	20d	Present results of all sensitivity analyses conducted to assess the robustness of the synthesized results.	N/A
Reporting biases	21	Present assessments of risk of bias due to missing results (arising from reporting biases) for each synthesis assessed.	40-42
Certainty of evidence	22	Present assessments of certainty (or confidence) in the body of evidence for each outcome assessed.	N/A
**DISCUSSION**			
Discussion	23a	Provide a general interpretation of the results in the context of other evidence.	45-48
	23b	Discuss any limitations of the evidence included in the review.	48-50
	23c	Discuss any limitations of the review processes used.	50-52
	23d	Discuss implications of the results for practice, policy, and future research.	52-53
**OTHER INFORMATION**			
Registration and protocol	24a	Provide registration information for the review, including register name and registration number, or state that the review was not registered.	7
	24b	Indicate where the review protocol can be accessed, or state that a protocol was not prepared.	7
	24c	Describe and explain any amendments to information provided at registration or in the protocol.	Appendix 3
Support	25	Describe sources of financial or non-financial support for the review, and the role of the funders or sponsors in the review.	1-2
Competing interests	26	Declare any competing interests of review authors.	1
Availability of data, code and other materials	27	Report which of the following are publicly available and where they can be found: template data collection forms; data extracted from included studies; data used for all analyses; analytic code; any other materials used in the review.	N/A

From: Page MJ, McKenzie JE, Bossuyt PM, Boutron I, Hoffmann TC, Mulrow CD, et al. The PRISMA 2020 statement: an updated guideline for reporting systematic reviews. BMJ 2021;372:n71. doi: 10.1136/bmj.n71

Synthesis Without Meta-analysis (SWiM) Reporting Items (Appendix 2)

The citation for the Synthesis Without Meta-analysis explanation and elaboration article is: Campbell M, McKenzie JE, Sowden A, Katikireddi SV, Brennan SE, Ellis S, Hartmann-Boyce J, Ryan R, Shepperd S, Thomas J, Welch V, Thomson H. Synthesis without meta-analysis (SWiM) in systematic reviews: reporting guideline BMJ 2020;368:l6890 http://dx.doi.org/10.1136/bmj.l6890

**SWiM is intended to complement and be used as an extension to PRISMA**			
**SWiM reporting item**	**Item description**	**Page in manuscript where item is reported**	**Other***
Methods			
**1** Grouping studies for synthesis	1a) Provide a description of, and rationale for, the groups used in the synthesis (e.g., groupings of populations, interventions, outcomes, study design)	13	
	1b) Detail and provide rationale for any changes made subsequent to the protocol in the groups used in the synthesis	N/A	
**2** Describe the standardised metric and transformation methods used	Describe the standardised metric for each outcome. Explain why the metric(s) was chosen, and describe any methods used to transform the intervention effects, as reported in the study, to the standardised metric, citing any methodological guidance consulted	11-12	
**3** Describe the synthesis methods	Describe and justify the methods used to synthesise the effects for each outcome when it was not possible to undertake a meta-analysis of effect estimates	11-13	
**4** Criteria used to prioritise results for summary and synthesis	Where applicable, provide the criteria used, with supporting justification, to select the particular studies, or a particular study, for the main synthesis or to draw conclusions from the synthesis (e.g., based on study design, risk of bias assessments, directness in relation to the review question)	N/A	
**5** Investigation of heterogeneity in reported effects	State the method(s) used to examine heterogeneity in reported effects when it was not possible to undertake a meta-analysis of effect estimates and its extensions to investigate heterogeneity	13	

**6** Certainty of evidence	Describe the methods used to assess certainty of the synthesis findings	N/A	
**7** Data presentation methods	Describe the graphical and tabular methods used to present the effects (e.g., tables, forest plots, harvest plots).<br>Specify key study characteristics (e.g., study design, risk of bias) used to order the studies, in the text and any tables or graphs, clearly referencing the studies included	13	
Results			
**8** Reporting results	For each comparison and outcome, provide a description of the synthesised findings, and the certainty of the findings. Describe the result in language that is consistent with the question the synthesis addresses, and indicate which studies contribute to the synthesis	16-40	
Discussion			
**9** Limitations of the synthesis	Report the limitations of the synthesis methods used and/or the groupings used in the synthesis, and how these affect the conclusions that can be drawn in relation to the original review question	51-52	

PRISMA=Preferred Reporting Items for Systematic Reviews and Meta-Analyses.
*If the information is not provided in the systematic review, give details of where this information is available (e.g., protocol, other published papers (provide citation details), or website (provide the URL)).

Changes to Protocol (Appendix 3)

Protocol reference: Emily Whitaker, Chloe Chessell, Maxwell Klapow, Cathy Creswell. A systematic review of guided, parent-led digital interventions for preadolescent children with emotional and behavioural problems. PROSPERO 2023 CRD42023484098.

Available from: https://www.crd.york.ac.uk/prospero/display_record.php?ID=CRD42023484098

The original version of the protocol was registered on PROSPERO in December 2023, prior to the database searches being conducted in January 2024. Once the title/abstract screening had commenced, it became apparent that clarifications were needed to the eligibility criteria in order to capture the type of studies needed to answer our research questions. Edits to the following sections of the protocol were made:

1) Exclusion criteria relating to the interventions:

- We clarified that the ‘guidance’ provided to parents as part of the intervention had to be more substantial than just technical assistance, and it should be provided by a human (rather than a chatbot or automated messages, for instance).

- We added a criterion to exclude interventions in which the only ‘digital’ element was that the intervention took place via videoconferencing software (such as Zoom or Microsoft Teams). Whilst use of videoconferencing software was acceptable to provide guidance to parents or as an aspect of the treatment, to be included in this review the intervention needed to also include a website, software or application that the parent uses themselves to access the treatment content.

2) Inclusion criteria relating to the participants:

- We extended the inclusion criteria relating to the child's emotional and/or behavioural problem (EBP), to reflect that in routine practice, the child will not always have a diagnosis or have been assessed with a standardised measure. Studies that take a pragmatic approach and determine that children have an EBP based on their usual assessment procedures in usual clinical practice, will be eligible for this review.

Search Strategy (Appendix 4)

From the original search conducted on 2nd January 2024.

Database	Search
PsycINFO	PsycINFO 1806 to present<br><br>1 (child* or preadolescen* or pre-adolescen* or pre-teen* or preteen* or youth* or paediatric* or pediatric* or juvenil* or offspring or young).mp. [mp=title, abstract, heading word, table of contents, key concepts, original title, tests & measures, mesh word] 1275767<br><br>2 (parent* or carer* or caregiver* or care-giver* or mother* or father* or family or families).mp. [mp=title, abstract, heading word, table of contents, key concepts, original title, tests & measures, mesh word] 792341<br><br>3 ((digital* or remote or computer* or online or web* or mobile or app or smartphone or virtual or cyber or internet or e-mental or emental or telehealth or tele-health) adj3 (intervention or treat* or program* or train* or support* or teach* or therap* or psychotherap*)).mp. [mp=title, abstract, heading word, table of contents, key concepts, original title, tests & measures, mesh word]52375<br><br>4 exp Digital Interventions/ 1700<br><br>5 (guid* or support* or led or assist*).mp. [mp=title, abstract, heading word, table of contents, key concepts, original title, tests & measures, mesh word] 1264864<br><br>6 ("emotion* problem*" or "emotion* disorder*" or "emotion* difficult*" or "behavio* problem*" or "behavio* disorder*" or "behavio* difficult*" or mental* or psychiatr* or psycho* or anxi* or depress* or mood or affective or internali* or worr* or fear* or obsessi* or compulsi* or OCD or panic or agoraphobi* or phobi* or inhibit* or shy* or mut* or "conduct problem*" or "conduct disorder*" or "conduct difficult*" or defian* or opposition* or "attention deficit" or inattenti* or externali* or ADHD or hyperactiv* or impuls* or disrupt* or aggress*).mp. [mp=title, abstract, heading word, table of contents, key concepts, original title, tests & measures, mesh word] 2790487<br><br>7 exp Mental Health/ 94543<br><br>8 exp Mental Disorders/ or exp Major Depression/ or exp Mental Health/ or exp Emotional Adjustment/ or exp Anxiety/ or exp Stress/ or exp Behavior Problems/ or exp Emotional Disturbances/ 1400375<br><br>9 3 or 4 52974

	10 6 or 7 or 8 3117285<br><br>11 1 and 2 and 5 and 9 and 10 1492<br><br>12 limit 11 to (human and english language) 1359
MEDLINE (Ovid)	Medline (Ovid MEDLINE® Epub Ahead of Print, In-Process & Other Non-Indexed Citations, Ovid MEDLINE® Daily and Ovid MEDLINE®) 1946 to present<br><br>1 (child* or preadolescen* or pre-adolescen* or pre-teen* or preteen* or youth* or paediatric* or pediatric* or juvenil* or offspring or young).mp. [mp=title, book title, abstract, original title, name of substance word, subject heading word, floating sub-heading word, keyword heading word, organism supplementary concept word, protocol supplementary concept word, rare disease supplementary concept word, unique identifier, synonyms, population supplementary concept word, anatomy supplementary concept word] 4210988<br><br>2 exp Child/ 2179907<br><br>3 (parent* or carer* or caregiver* or care-giver* or mother* or father* or family or families).mp. [mp=title, book title, abstract, original title, name of substance word, subject heading word, floating sub-heading word, keyword heading word, organism supplementary concept word, protocol supplementary concept word, rare disease supplementary concept word, unique identifier, synonyms, population supplementary concept word, anatomy supplementary concept word] 2085122<br><br>4 ((digital* or remote or computer* or online or web* or mobile or app or smartphone or virtual or cyber or internet or e-mental or emental or telehealth or tele-health) adj3 (intervention or treat* or program* or train* or support* or teach* or therap* or psychotherap*)).mp. [mp=title, book title, abstract, original title, name of substance word, subject heading word, floating sub-heading word, keyword heading word, organism supplementary concept word, protocol supplementary concept word, rare disease supplementary concept word, unique identifier, synonyms, population supplementary concept word, anatomy supplementary concept word] 96945<br><br>5 Internet-Based Intervention/ 1182<br><br>6 Digital Technology/ 804<br><br>7 (guid* or support* or led or assist*).mp. [mp=title, book title, abstract, original title, name of substance word, subject heading word, floating sub-heading word, keyword heading word, organism supplementary concept word, protocol supplementary concept word, rare disease supplementary concept word, unique

	identifier, synonyms, population supplementary concept word, anatomy supplementary concept word] 13242842<br><br>8 ("emotion* problem*" or "emotion* disorder*" or "emotion* difficult*" or "behavio* problem*" or "behavio* disorder*" or "behavio* difficult*" or mental* or psychiatr* or psycho* or anxi* or depress* or mood or affective or internali* or worr* or fear* or obsessi* or compulsi* or OCD or panic or agoraphobi* or phobi* or inhibit* or shy* or mut* or "conduct problem*" or "conduct disorder*" or "conduct difficult*" or defian* or opposition* or "attention deficit" or inattenti* or externali* or ADHD or hyperactiv* or impuls* or disrupt* or aggress*).mp. [mp=title, book title, abstract, original title, name of substance word, subject heading word, floating sub-heading word, keyword heading word, organism supplementary concept word, protocol supplementary concept word, rare disease supplementary concept word, unique identifier, synonyms, population supplementary concept word, anatomy supplementary concept word] 7811377<br><br>9 Mental Health/ 64548<br><br>10 Mental Disorders/ or Affective Symptoms/ or Child Behavior Disorders/ or Anxiety/ 314489<br><br>11 Problem Behavior/ or Attention Deficit Disorder with Hyperactivity/ 39158<br><br>12 "Disruptive, Impulse Control, and Conduct Disorders"/ 2728<br><br>13 Mood Disorders/ 16102<br><br>14 1 or 2 4210988<br><br>15 4 or 5 or 6 97612<br><br>16 8 or 9 or 10 or 11 or 12 or 13 7811628<br><br>17 3 and 7 and 14 and 15 and 16 1936<br><br>18 limit 17 to (english language and humans) 1575
Embase	Embase 1974 to present<br><br>1 (child* or preadolescen* or pre-adolescen* or pre-teen* or preteen* or youth* or paediatric* or pediatric* or juvenil* or offspring or young).mp. [mp=title, abstract, heading word, drug trade name, original title, device manufacturer, drug manufacturer, device trade name, keyword heading word, floating subheading word, candidate term word] 4512876

	2 child/ 2140793<br><br>3 (parent* or carer* or caregiver* or care-giver* or mother* or father* or family or families).mp. [mp=title, abstract, heading word, drug trade name, original title, device manufacturer, drug manufacturer, device trade name, keyword heading word, floating subheading word, candidate term word] 2566383<br><br>4 ((digital* or remote or computer* or online or web* or mobile or app or smartphone or virtual or cyber or internet or e-mental or emental or telehealth or tele-health) adj3 (intervention or treat* or program* or train* or support* or teach* or therap* or psychotherap*)).mp. [mp=title, abstract, heading word, drug trade name, original title, device manufacturer, drug manufacturer, device trade name, keyword heading word, floating subheading word, candidate term word] 291793<br><br>5 web-based intervention/ 3025<br><br>6 digital technology/ 4911<br><br>7 (guid* or support* or led or assist*).mp. [mp=title, abstract, heading word, drug trade name, original title, device manufacturer, drug manufacturer, device trade name, keyword heading word, floating subheading word, candidate term word] 6463870<br><br>8 ("emotion* problem*" or "emotion* disorder*" or "emotion* difficult*" or "behavio* problem*" or "behavio* disorder*" or "behavio* difficult*" or mental* or psychiatr* or psycho* or anxi* or depress* or mood or affective or internali* or worr* or fear* or obsessi* or compulsi* or OCD or panic or agoraphobi* or phobi* or inhibit* or shy* or mut* or "conduct problem*" or "conduct disorder*" or "conduct difficult*" or defian* or opposition* or "attention deficit" or inattenti* or externali* or ADHD or hyperactiv* or impuls* or disrupt* or aggress*).mp. [mp=title, abstract, heading word, drug trade name, original title, device manufacturer, drug manufacturer, device trade name, keyword heading word, floating subheading word, candidate term word] 9708743<br><br>9 mental health/ 215974<br><br>10 behavior disorder/ or emotional disorder/ 78598<br><br>11 problem behavior/ 9554<br><br>12 anxiety/ or social anxiety/ or anxiety disorder/ or generalized anxiety disorder/ or separation anxiety/ or school anxiety/ 392089<br><br>13 mood disorder/ 54273<br><br>14 attention deficit disorder/ or attention deficit hyperactivity disorder/ 79344

	15 1 or 2 4512876<br><br>16 4 or 5 or 6 295738<br><br>17 8 or 9 or 10 or 11 or 12 or 13 or 14 9709492<br><br>18 3 and 7 and 15 and 16 and 17 2468<br><br>19 limit 18 to (human and english language) 2363
Web of Science	child* OR preadolescen* OR pre-adolescen* OR pre-teen* OR preteen* OR youth* OR paediatric* OR pediatric* OR juvenil* OR offspring OR young (Topic) and parent* OR carer* OR caregiver* OR care-giver* OR mother* OR father* OR family OR families (Topic) and (digital* OR remote OR computer* OR online OR web* OR mobile OR app OR smartphone OR virtual OR cyber OR internet OR e-mental OR emental OR telehealth OR tele-health) NEAR/3 (intervention OR treat* OR program* OR train* OR support* OR teach* OR therap* OR psychotherap*) (Topic) and guid* OR support* OR led OR assist* (Topic) and “emotion* problem*” OR “emotion* disorder*” OR “emotion* difficult*” OR “behavio* problem*” OR “behavio* disorder*” OR “behavio* difficult*” OR mental* OR psychiatr* OR psycho* OR anxi* OR depress* OR mood OR affective OR internali* OR worr* OR fear* OR obsessi* OR compulsi* OR OCD OR panic OR agoraphobi* OR phobi* OR inhibit* OR shy* OR mut* OR “conduct problem*” OR “conduct disorder*” OR “conduct difficult*” OR defian* OR opposition* OR “attention deficit” OR inattenti* OR externali* OR ADHD OR hyperactiv* OR impuls* OR disrupt* OR aggress* (Topic) and English (Languages)
Scopus	(TITLE-ABS-KEY (child* OR preadolescen* OR pre-adolescen* OR pre-teen* OR preteen* OR youth* OR paediatric* OR pediatric* OR juvenil* OR offspring OR young) AND TITLE-ABS-KEY (parent* OR carer* OR caregiver* OR care-giver* OR mother* OR father* OR family OR families) AND TITLE-ABS-KEY ((digital* OR remote OR computer* OR online OR web* OR mobile OR app OR smartphone OR virtual OR cyber OR internet OR e-mental OR emental OR telehealth OR tele-health) W/3 (intervention OR treat* OR program* OR train* OR support* OR teach* OR therap* OR psychotherap*)) AND TITLE-ABS-KEY (guid* OR support* OR led OR assist*) AND TITLE-ABS-KEY ("emotion* problem*" OR "emotion* disorder*" OR "emotion* difficult*" OR "behavio* problem*" OR "behavio* disorder*" OR "behavio* difficult*" OR mental* OR psychiatr* OR psycho* OR anxi* OR depress* OR mood OR affective OR internali* OR worr* OR fear* OR obsessi* OR compulsi* OR ocd OR panic OR agoraphobi* OR phobi* OR inhibit* OR shy* OR mut* OR "conduct problem*" OR "conduct disorder*" OR "conduct difficult*" OR defian* OR opposition* OR "attention deficit" OR inattenti* OR externali* OR adhd OR hyperactiv* OR impuls* OR disrupt* OR aggress*)) AND (LIMIT-TO (LANGUAGE , "English")) AND (LIMIT-TO (EXACTKEYWORD , "Human"))

Eligibility Criteria (Appendix 5)

Inclusion criteria

A study was eligible for inclusion in the review if it met all of the following criteria:

(1) The full text was available in English.

(2) The study evaluated the effect of a psychological intervention in which a parent is supported to deliver the intervention by a facilitator.

(3) The intervention was targeted at children with at least one emotional and/or behavioural problem (EBP). Children should be assessed as having an EBP via at least one of the following methods:

(a) a formal diagnosis

(b) elevated symptoms according to an established measure

(c) a clinical assessment/decision by a clinical team.

(4) The intervention was fully ‘digital’. For the purposes of this review, this was classed as an intervention where the content is delivered via at least one of the following methods:

(a) the internet (e.g. a website)

(b) a smartphone (e.g. an app or a mobile website)

(c) software.

(5) The participants were parents/carers of children aged 4-12 years old. Studies with a wider age range (up to 14 years old) were included if the mean age was 4-12 years old.

(6) The study reported quantitative data from at least pre- and post-intervention outcome measures of symptoms/interference related to the child’s EBP.

Exclusion criteria

A study was excluded from the review if it met any of the following:

(1) The intervention involved any face-to-face contact with the facilitator.

(2) The intervention is not directly delivered via the parent.

(3) The intervention is delivered directly to the child.

(4) The intervention involved any physical materials (i.e. the intervention was not fully digital as these materials were a necessary aspect of the intervention).

(5) The intervention did not feature any guidance by a facilitator (i.e. the intervention should not be purely self-help). For the purposes of this review, 'guidance' had to go beyond just technical assistance or encouragement/checking the parent is completing the intervention.

(6) The study was a systematic review, meta-analysis or protocol.

(7) The study reported previously published data. In cases of studies that reported on new data using the same sample of participants, the data were pooled and the studies combined for the purposes of this review.

Full Text Stepped Exclusion List (Appendix 6)

Studies were excluded at the full text stage in a stepped fashion; that is, the first criterion that the study failed to meet was recorded as the study's exclusion reason.

(1) Study is a review, meta-analysis or protocol

(2) Ineligible mean age or age range

(3) Intervention is not digital

(4) Intervention is not parent-led

(5) Intervention is not guided

(6) Intervention does not target child's EBP

(7) Child does not meet EBP criteria

(8) Outcomes are not measures of child's E/B symptoms/interference levels

(9) No pre-/post-intervention outcome measures

(10) Uses previously published data

Full Text Exclusion Reasons (Appendix 7)

The exclusion reasons for the 271 studies screened out at the full text stage of the database searches are recorded below.

(1) Excluded as the paper is a review, meta-analysis or protocol *(n = 1)*

Dopfner 2020

(2) Excluded as the participants' mean age or age range was ineligible *(n = 49)*

Under this criterion, studies were excluded if:

- The children's mean age was 3 years or younger, or 13 years or older.
- The children's upper age range was 15 years or older.

Aspvall 2020
Bell 2025
Blanchet 2024
Boydston 2020
Boydston 2021
Clarke 2014
Dai 2023
Davidson 2019
Day 2018
Fisher 2020
Fleming 2020
Gerow 2023
Hodges 2022
Hails 2025
Holtrop 2023
Huffman 2023
Ingersoll 2016
Ingersoll 2023
Johnson 2014
Johnson 2014
Jurigova 2021
Law 2018
Lawrence-Sidebottom 2023
Lo 2024
Lu 2024
Minder 2018
Moor 2019
Nicksic-Springer 2016
Pickard 2016
Piotrowska 2020
Qu 2023
Rendek 2008
Rooks-Ellis 2020
Sadeghi 2022
Schniering 2022
Shahnavaz 2018
Spigner 2016
Stasiak 2018
Stewart 2017
Stewart 2020
Sullivan 2019
Vismara 2013
Vismara 2018
Vreeken-Ross 2022
Wade 2004
Wade 2005
Yap 2018
Yap 2019
Yi 2021

(3) Excluded as the intervention was not classed as digital *(n = 80)*

Under this criterion, studies were excluded if:

- The only 'digital' aspect was that the intervention was conducted via videoconferencing.
- The guidance or another element of the intervention was face-to-face.
- The intervention involved physical materials (e.g. paper workbooks/manuals).

Agazzi 2022
Altafim 2024
Andrews 2020
Druskin 2023
DuPaul 2018
El-Khani 2021
Javier 2023
Javier 2025
Jewell 2023
Paiva 2024
Pogue 2023
Rabbitt 2016

Andrews 2022
Bedard 2023
Benson 2018
Bono 2022
Boutain 2015
Boutain 2020
Breider 2019
Breider 2024
Byrne 2022
Candan 2023
Comer 2021
Craig 2023
Craig 2024
Cummings 2008
Demily 2016
Diaz-Stransky 2020
Díez-Juan 2014
Emadian 2016
Engelbrektsson 2023
Fleming 2017
Fogarty 2022
Gewirtz 2014
Ghodrat 2022
Gould 2023
Guzick 2022
Guzick 2024
Hall 2020
Hall 2022
Hartman 2000
Hayford 2022
Holzman 2023
Hong 2023
Inoue 2024
James Riegler 2020
Jimenez-Gomez 2023
Jouen 2017
Kolb 2007
Lau 2022
Laughlin 2021
Lindgren 2016
Lyneham 2006
Malcolm 2021
Marino 2020
Marino 2022
Mast 2014
McCall 2023
Merrill 2023
Miller-Johnson 2022
Mogil 2022
Munneke 2001
Narzisi 2020
Sanders 2008
Scott 2001
Shelleby 2015
Smith 2023
Speidel 2023
Storch 2015
Taylor 2008
Tomlinson 2021
Tse 2015
Tuntipuchitanon 2022
Wacker 2013
Wade 2009
Ward 2022
Wellington 2022
Whaley 2020
Yamanaka 2023
Zhang 2023

(4) Excluded as the intervention was not classed as parent-led *(n = 64)*

Under this criterion, studies were excluded if:

- The child was involved in any aspect of the intervention.

Amirova 2023
Aspvall 2018
Bechor 2014
Bikic 2018
Blocher 2013
Bul 2016
Chacko 2014
Chu 2023
Comer 2014
Comer 2017
Conaughton 2017
Crawford 2013
delaGuia 2013
Epstein 2016
Gallen 2021
Goertz-Dorten 2022
Grunewaldt 2013
Grunewaldt 2016
Ha 2022
Haack 2023
Healy 2023
Houck 2022
Jolstedt 2018
Jolstedt 2021
Khanna 2010
Ko 2002
Ko 2003
Kollins 2020
Lalouni 2017
Lalouni 2019
Lathren 2023
Lee 2022
Lim 2010
Lim 2019
Lomas 2002
March 2019
Maskey 2014
McCashin 2022
McDermott 2020
McLellan 2024
Minder 2019
Neely 2016
Orgiles 2023
Pettit 2017
Sarver 2014
Shalev 2007
Shimek 2012
Sourander 2025
Spaniol 2021
Steiner 2011
Tan 2022
Thomson 2019
Treves 2023
Vigerland 2013
Vigerland 2015
Vigerland 2016
Vigerland 2017
Vigerland 2022
Voss 2019
Vries 2015
Wainer 2011
Wells 2021
Wiguna 2021
Yamanaka 2024

(5) Excluded as the intervention was not classed as guided *(n = 34)*

Under this criterion, studies were excluded if:

- The intervention did not feature any guidance from a facilitator.
- The guidance provided was only to offer technical assistance or encouragement.

Baker 2017	Keller 2009	Nunes 2022	Sim 2020
Blackman 2020	Kenworthy 2023	Owen 2018	Sim 2022
Candelari 2023	Khanna 2017	Penev 2021	Suarez 2018
Chesnut 2020	Kishida 2022	Pokorna 2024	Trudel 2021
DeGarmo 2019	Lee 2024	Prinz 2022	Tully 2024
DiFonte 2022	Mackenzie 1998	Sanders 2012	Wendelken 2023
Donkin 2023	MacKenzie 1999	Sanders 2014	Yam-Ubon 2023
Feil 2018	Metcalfe 2022	Sapru 2018	
Hutchings 2023	Nathanson 2022	Siljeholm 2022	

(6) Excluded as the intervention did not target the child's EBP *(n = 8)*

Under this criterion, studies were excluded if:

- The intervention was not primarily aimed at addressing symptoms related to the child's EBP.

Cernvall 2015	Colucci 2022	David 2017	Whittingham 2022
Cernvall 2017	Connell 2023	Metcalfe 2021	Zayde 2022

(7) Child did not meet EBP criteria *(n = 16)*

Under this criterion, studies were excluded if:

- The child was either

  (a) not formally diagnosed with an EBP,

  (b) not displaying elevated symptoms of an EBP according to an established measure,

  (c) or not considered to have an EBP following a clinical assessment.

Aguilar 2019	Havighurst 2024	Mauricio 2024	Stormshak 2019
Antonini 2014	Inoue 2022	Morgan 2016	Stormshak 2024
Burek 2021	Lester 2024	Morgan 2017	Togashi 2023
Connell 2024	Mast 2012	Pennefather 2018	Waligorska 2012

(8) Outcomes are not measures of child's E/B symptoms *(n = 6)*

Under this criterion, studies were excluded if:

- The outcomes did not measure symptoms or interference related to the EBP that the child was receiving the intervention for.
- The outcomes were all related to usability or acceptability.
- The outcomes related to the parent's mental health.

Fisher 2024	Mazenc 2017	Ristkari 2019
Lintula 2024	Morgan 2018	Wähnke 2024a

(9) No pre- / post-intervention outcome measures *(n = 5)*

Under this criterion, studies were excluded if:

- The outcome measures were suitable but not administered at appropriate timepoints.
- The study did not actually test the intervention so outcome measures were not pre-post.

Dittman 2014	Ortiz-Becher 2005	Wähnke 2024b
Liverpool 2021	Park 2020	

(10) Uses previously published data *(n = 8)*

Under this criterion, studies were excluded if:

- No new data was included with regards to pre-post intervention measures.

Engelbrektsson 2023	Hogstrom 2013	Hogstrom 2022
Fossum 2018	Hogstrom 2014	Sonuga-Barke 2023

- Plus studies that reported follow-up data only that will be pooled with included studies.*

Hogstrom 2015	Sourander 2018

* In addition to the two pooled follow-up studies from the database searches above, a third eligible follow-up study (Sourander 2024) was identified in the updated forward citation searching.

Examples of excluded studies that may appear to meet the eligibility criteria (from both the database searches and the hand searching):

- **Morgan (2016):** children were "temperamentally inhibited", which we did not consider to meet the criteria for an EBP for the purposes of this review.
- **Diaz-Stransky (2020):** parents received in-person support to initially access the intervention and be introduced to elements of the treatment, and so we did not consider the intervention to be fully digital.
- **Sim (2020):** the guidance is explicitly stated as being "not to provide individual parenting or clinical consult", which we did not consider to be adequate guidance for the purposes of this review.
- **McLellan (2024):** parents complete the intervention alongside their child, which came under the exclusion criterion of "The intervention is delivered directly to the child".
- **Wähnke (2024):** the paper focused on links between usage and symptom change, but did not report the outcome data for the arm with therapist support and was therefore excluded under the "No pre- / post-intervention outcome measures" criterion. The main trial outcome paper that this study was based on has not been published at the time of writing.
- **Sourander (2025):** children were directly involved in the treatment so the intervention was excluded for not being parent-led.

Sample Characteristics – Further Details (Appendix 8)

First author (date); intervention	Sample size (intervention)	Mean child age in years (SD)	Child gender	Child ethnicity	Child diagnostic criteria	Mean parent age in years (SD)	Parent gender	Parent ethnicity	Family socio-economic status (parent education and/or household income)
Mazenc (2021) [a]; ACE	93 / 47	9.38 (1.67)	57.4% female; 42.6% male	85.1% White; 4.3% Arab; 4.3% Asian; 2.3% Indigenous; 2% Black	SCAS-8 during screening	39.81 (4.76)	93.6% female	NR	Parent education: 6.4% high school diploma or equivalent; 36.2% undergraduate degree; 29.8% graduate degree; 27.6% technical/trade school or some university<br><br>Household income: 19.2% < $60,000; 25.5% $60,000-$100,000; 53.2% > $100,000
Donovan (2014) [b]; BRAVE-ONLINE	23	3.96 (0.64) *	52.2% female; 47.8% male *	94.2% born in Australia; 1.9% born in England; 1.9% born in Ireland; 1.9% born in South Africa **	Primary diagnosis of anxiety (social, separation or generalised anxiety disorder, or specific phobia)	Mothers: 36.33 (4.83); fathers: 38.37 (6.28) * **	Mostly mothers * **	NR	Household income: 5.8% $21,000-$40,000; 9.6% $41,000-$60,000; 15.4% $61,000-$80,000; 13.5% $81,000-$100,000; 55.8% over $100,000 **
Creswell (2024); OSI	222	9.31 (1.83)	57% female; 41% male; 1% other; 1% prefer not to say	87% White; 9% mixed; 1% Asian or Asian British; <1% Black or Black British; 1% other ethnic groups; 1% not stated	Assessment by clinical team as part of routine practice	39 (5.93)	96% female, 4% male	91% White; 5% mixed; 1% Asian or Asian British; <1% Black or Black British; 1% other ethnic groups; 1% not stated	Parent education: 16% school completion; 46% further education; 18% higher education; 20% postgraduate education.<br><br>Household income (per year): 12% <£16,000; 19% £16,001-£30,000; 10% £30,001-£40,000; 8% £40,001-£50,000; 9% £50,001-£60,000; 8% £60,001-£70,000; 6% £70,001-£80,000; 4% £80,001-£90,000; 6% £90,001-£120,000; 2% >£120,000; 17% prefer not to say

Green (2023) [c]; OSI	40	8.88 (0.38) *	65% female; 35% male *	79% White British; 11% any other White background; 6% any other ethnic background; 4% not stated	SCAS-8 (study 1) / iCATS-2 (study 2)	41.57 (3.83) *	90% female; 7.5% male; 2.5% prefer not to say *	NR	Parent education: 12% school completion; 39% further education; 50% higher/postgraduate education
Hill (2022); OSI	23	9.65 (1.19)	73.9% female	65.2% White British; 34.8% unknown	Assessment by clinical team as part of routine practice	NR	95.6% mothers; 4.3% fathers	NR	NR
Poetar (2024); ParentKIT	21	10.86 (2.43)	66.7% female	NR	Emotional problems subscale of SDQ	40.43 (3.01)	81% female	NR	Parent education: 9.5% high school; 42.9% bachelor's degree; 47.6% master's degree
Dadds (2019) [d]; AccessEI	66 / 35	6.79 (1.68) / 7.49 (2.92)	22.4% female; 77.6% male / 20% female; 80% male	NR	Met full diagnosis for CD or ODD according to DSM-IV or displayed subclinical symptoms	NR	46% both mother and father; 49% mother only; 3% father only; 2% other * [e]	NR	Mother's education: 27.27% less than or equal to year 10; 48.48% year 12 or equivalent; 24.24% university. Father's education level: 36.92% less than or equal to year 10; 57.89% year 12 or equivalent; 12.31% university.<br><br>Household income: 24.39% less than 40k; 29.27% 41k-70k; 19.51% 71k-100k; 12.2% 101k-130k; 0% 131k-160k; 7.32% more than 160k / Mother's education: 0% less than or equal to year 10; 8.57% year 12 or equivalent; 91.43% university. Father's education level: 5.88% less than or equal to year 10; 38.24% year 12 or equivalent; 55.88% university. Household income: 28.57% less than 40k; 34.29% 41k-70k; 22.86% 71k-100k; 14.29% 101k-130k; 0% 131k-160k; 8.57% more than 160k

Enebrink (2012); iCOMET	58	6.71 (2.31)	46.6% female, 53.4% male	98.3% born in Sweden	One SD above the mean on the ECBI	NR	69.2% both mother and father; 27.9% mother only; 2.9% father only **	NR	Mother's education: 72.4% were university educated. Father's education: 56.9% were university educated.
Ghaderi (2018); iCOMET	109	NR	NR	NR	Score 3 or more on the conduct subscale of the SDQ	NR	NR	NR	Parent education: 9.2% primary school; 22.9% high school (2 years); 24.8% high school (3-4 years); 43.1% college/university<br><br>Household income: 11% insufficient related to expenses; 30.3% almost sufficient; 52.3% sufficient: we don't worry; 6.4% good: don't think of expenses
A. Sourander (2016); SFSW	232	4	37.3% female, 62.7% male	NR	Score 5 or more on the conduct subscale of the SDQ	Mothers: 30.5 (NR); fathers: 33.2 (NR)	NR	NR	Mother's education: 5.7% elementary school or less; 37% secondary education; 57.4% college or university degree. Father's education: 7.4% elementary school or less; 45.8% secondary education; 46.8% college or university degree.
A. Sourander (2022); SFSW	600	4	39.7% female; 60.3% male	NR	Score 5 or more on the conduct subscale of the SDQ	Mothers: 30.3 (NR); fathers: 32.7 (NR)	NR	NR	Mother's education: 2.5% elementary school or less; 34.2% secondary education; 63.3% college or university degree. Father's education: 4.8% elementary school or less; 50.1% secondary education; 45.1% college or university degree.
S. Sourander (2022); SFSW	50	NR	26% female; 74% male	NR	Conduct subscale of the SDQ	Mothers: 31.9 (NR); fathers: 32.8 (NR)	NR	NR	Mother's education: 22% secondary education; 76% college or university degree; 2% other. Father's education: 7% elementary school or less; 24% secondary education; 7% college or university degree; 2% other.

Franke (2020) **; Triple P Online	27	4 (NR)	71.7% male	79.2% New Zealand European ethnicity	Met cut off on WWP and PACS	35.4 (4.87)	100% mothers but some (*n* = 43) fathers also took part	NR	Mother's education: 55.7% of mothers had a university degree<br><br>Household income: one third of families had an annual income below NZ$75,000; one third above NZ$100,000

***** *Data provided by author. ** Data includes the control arm rather than just the intervention arm.* ***NR*** *= not reported.* **CD =** *conduct disorder.* **ODD =** *oppositional defiant disorder.* ***Digital intervention abbreviations:*** *ACE = Anxiety treatment for Children through online Education; OSI = Online Support and Intervention for child anxiety; SFSW = Strongest Families Smart Website.* ***Diagnostic measure abbreviations:*** *SCAS-8 = Spence Children's Anxiety Scale; SDQ = Strengths and Difficulties Questionnaire; ECBI = Eyberg Child Behavior Inventory; DBD = Disruptive Behavior Disorders Rating Scale; WWP = Werry–Weiss–Peters activity rating scale; PACS = Parental Account of Child Symptoms.*

[a] ***Mazenc (2021)****: whilst 93 families started the intervention, demographics were only reported for the 47 completers.* [b] ***Donovan (2014):*** *although the mean age for the intervention arm was just under 4 years old, this study was included in review as the overall mean age was 4.08 years. The parent demographics relate to the whole sample.* [c] ***Green (2023):*** *47 families received the intervention across both studies, however only the second study met the inclusion criteria for this review, which had 40 participants. Child ethnicity and family socio-economic status refer to the full sample rather than the 40 children.* [d] ***Dadds (2019)****: the forward slash reflects Study 1 / Study 2. Where no forward slash is used, the information was the same across both studies.* [e] ***Dadds (2019):*** *Parent gender was calculated by the review authors using data provided by the study author, and relates to the intervention groups of both studies combined.*

Quality Assessment (Appendix 9)

Criteria

1. Question/objective sufficiently described?

2. Study design evident and appropriate?

3. Method of subject/comparison group selection or source of information/input variables described and appropriate?

4. Subject (and comparison group, if applicable) characteristics sufficiently described?

5. If interventional and random allocation was possible, was it described?

6. If interventional and blinding of investigators was possible, was it reported?

7. If interventional and blinding of subjects was possible, was it reported?

8. Outcome and (if applicable) exposure measure(s) well defined and robust to measurement / misclassification bias? Means of assessment reported?

9. Sample size appropriate?

10. Analytic methods described/justified and appropriate?

11. Some estimate of variance is reported for the main results?

12. Controlled for confounding?

13. Results reported in sufficient detail?

14. Conclusions supported by the results?

Scoring: Yes = 2 points; Partial = 1 point; No = 0 points. Not applicable (N/A) is also an option.
Taken from Kmet et al. (2004), available via https://www.ihe.ca/download/standard_quality_assessment_criteria_for_evaluating_primary_research_papers_from_a_variety_of_fields.pdf

Examples of how the criteria were applied to the included studies

General notes that the authors applied to most criterions, to clarify those outlined in the Kmet et al. guidance (linked above), are explained in italics.

1. Question / objective sufficiently described?

General note: studies score full points as long as a research question, objective and/or hypothesis were mentioned at some point in the introduction or early on in the methods section.

2. Study design evident and appropriate?

General note: randomised controlled trials (RCTs) had to provide explicit further information beyond just stating that they were an RCT (for example, inferiority, blinded, number of conditions).

- Poetar et al. (2024) were rated 'partial' as although the study is identified as a randomised controlled trial in the title, there is no 'Design' section or similar containing further details about the type of RCT.
- Ghaderi et al. (2018) were rated 'partial' as the study was not explicitly identified as a randomised controlled trial until the discussion.

4. Subject (and comparison group, if applicable) characteristics sufficiently described?

General note: studies did not score the full two points (i.e., a 'yes') if no parent gender or age details were given – we considered these to be key demographics for a parent-led intervention.

- Donovan et al. (2014) were rated 'partial' as the sample demographics were not broken down by arm, and age/gender of parents were missing (although these details were provided by the authors on request).
- Ghaderi et al. (2018) were rated 'partial' as although some demographics information was outlined, key child demographics (gender and mean age) were missing, along with no details about the age/gender of the parents.

5. If interventional and random allocation was possible, was it described?

General note: we marked this criterion as 'not applicable' for studies without a control group.

- Dadds et al. (2019) were rated 'partial' as although the randomisation procedure was described in Study 1, there was no mention of the procedure in Study 2, and it was therefore not clear whether the procedure was the same across both studies.
- Franke et al. (2020) were rated 'partial' as randomisation was mentioned, but the method of randomisation was not described.

6. If interventional and blinding of investigators was possible, was it reported?

General note: studies had to give some indication of which job roles or aspects of the study were blinded in order to score the full two points (i.e., a 'yes').

- Creswell et al. (2024) were rated 'yes' it was stated that the research assistants were not blinded to participant allocation, but the statisticians were.
- Poetar et al. (2024) were rated 'yes' as it was stated that the randomisation procedure was conducted by an independent researcher who was not involved in the study.

7. If interventional and blinding of subjects was possible, was it reported?

General note: we took the decision to mark this criterion as 'not applicable' for all studies, as given the nature of digital intervention, we did not consider the blinding of participants to be possible.

9. Sample size appropriate?

- Enebrink et al. (2012) were rated 'partial' as although the sample sizes appeared adequate, there was no mention of a power calculation or any justification for the sample size.
- Ghaderi et al. (2018) and Franke et al. (2020) were both rated 'partial' as although a power analysis was done, the studies were under powered due to not reaching their recruitment targets.
- Mazenc (2021) was rated as 'partial' as although a power analysis was done, significant drop out from the intervention results in the study being underpowered.

10. Analytic methods described/justified and appropriate?

General note: studies were unable to be rated 'Yes' if they only conducted/reported completer analyses (as opposed to intent-to-treat), given that only reporting on those who completed the intervention may skew the outcomes (Andrade, 2022).
